# Diverse Gene Regulatory Mechanisms Alter Rattlesnake Venom Gene Expression at Fine Evolutionary Scales

**DOI:** 10.1093/gbe/evae110

**Published:** 2024-05-16

**Authors:** Siddharth S Gopalan, Blair W Perry, Yannick Z Francioli, Drew R Schield, Hannah D Guss, Justin M Bernstein, Kaas Ballard, Cara F Smith, Anthony J Saviola, Richard H Adams, Stephen P Mackessy, Todd A Castoe

**Affiliations:** Department of Biology, University of Texas at Arlington, Arlington, TX 76019, USA; Department of Biology, University of Texas at Arlington, Arlington, TX 76019, USA; School of Biological Sciences, Washington State University, Pullman, WA 99164, USA; Department of Biology, University of Texas at Arlington, Arlington, TX 76019, USA; Department of Biology, University of Virginia, Charlottesville, VA 22903, USA; Department of Biology, University of Texas at Arlington, Arlington, TX 76019, USA; Department of Biology, University of Texas at Arlington, Arlington, TX 76019, USA; Department of Biology, University of Texas at Arlington, Arlington, TX 76019, USA; Department of Biochemistry and Molecular Genetics, University of Colorado Denver, Aurora, CO 80045, USA; Department of Biochemistry and Molecular Genetics, University of Colorado Denver, Aurora, CO 80045, USA; Department of Entomology and Plant Pathology, University of Arkansas Agricultural Experimental Station, University of Arkansas, Fayetteville, AR 72701, USA; Department of Biological Sciences, University of Northern Colorado, Greeley, CO 80639, USA; Department of Biology, University of Texas at Arlington, Arlington, TX 76019, USA

**Keywords:** ATAC-seq, chromatin, cis-regulatory element, CTCF, enhancer, gene regulatory networks

## Abstract

Understanding and predicting the relationships between genotype and phenotype is often challenging, largely due to the complex nature of eukaryotic gene regulation. A step towards this goal is to map how phenotypic diversity evolves through genomic changes that modify gene regulatory interactions. Using the Prairie Rattlesnake (*Crotalus viridis*) and related species, we integrate mRNA-seq, proteomic, ATAC-seq and whole-genome resequencing data to understand how specific evolutionary modifications to gene regulatory network components produce differences in venom gene expression. Through comparisons within and between species, we find a remarkably high degree of gene expression and regulatory network variation across even a shallow level of evolutionary divergence. We use these data to test hypotheses about the roles of specific trans-factors and cis-regulatory elements, how these roles may vary across venom genes and gene families, and how variation in regulatory systems drive diversity in venom phenotypes. Our results illustrate that differences in chromatin and genotype at regulatory elements play major roles in modulating expression. However, we also find that enhancer deletions, differences in transcription factor expression, and variation in activity of the insulator protein CTCF also likely impact venom phenotypes. Our findings provide insight into the diversity and gene-specificity of gene regulatory features and highlight the value of comparative studies to link gene regulatory network variation to phenotypic variation.

**Significance**

The breath of factors involved in the regulation of eukaryotic genes makes it challenging to quantify their individual contributions to gene expression differences, and to identify genomic mechanisms that give rise to phenotypic variation. Here, we address this challenge by leveraging naturally existing regulatory and phenotypic variation in snake venom systems across a closely related group of rattlesnakes. Across venom genes and gene families, we find that variation in chromatin and genotype at regulatory elements play dominant roles in modulating expression. Our results provide new perspectives on the extent of standing variation that may impact gene regulatory function even at shallow evolutionary divergences in a highly adaptive trait, highlighting the diversity and specificity of the genomic mechanisms that may underlie such variation.

## Introduction

Understanding how phenotypes evolve through genomic changes that modify gene regulatory interactions is central to understanding the basis of organismal diversity, and for linking variation in genotype to phenotype (Crombach and Hogeweg 2008; Romero et al. 2012; Wittkopp and Kalay 2012). However, the complexity of eukaryotic gene regulation poses many challenges for inferring how genomic variation manifests in phenotypic variation. Differences in gene expression can be driven by synergistic contributions of a variety of factors, including differences in transcription factor (TF) expression or activation (Spitz and Furlong 2012), differences in chromatin state that modulates access to cis-regulatory elements (CREs) (Buenrostro et al. 2015), variation in genotype at cis*-*elements that impacts TF binding (Rockman and Wray 2002; Wittkopp and Kalay 2012), and the activity of noncoding RNAs (Zheng et al. 2023). Studying how gene regulatory networks (GRNs) evolves to modulate expression of phenotypes across populations and species has the potential to provide new insights into the regulatory roles these factors play and thus provide a framework for linking regulatory network variation with trait variation. There are, however, few examples that provide baseline expectations for the relative contributions of chromatin accessibility changes, trans-factor differences, or sequence variation at CREs to gene expression differences at fine scales, such as between populations or among closely related species (e.g. Edsall et al. 2019; Barr et al. 2023). Accordingly, our understanding of which components of GRNs play predominant roles in generating gene expression differences at such fine scales, and how this regulatory architecture varies across genes, remains incomplete.

Snake venom provides a powerful system to map the relationships between genotypic, regulatory, and phenotypic variation due to the number of distinct venom gene families that contribute proteins to venom (Mackessy 2010; Tasoulis and Isbister 2017; Schield et al. 2019a; Casewell et al. 2020; Zancolli and Casewell 2020; Mackessy 2021). The diversity of venom composition across populations and species also provides comparative power to study evolutionary change at shallow scales of evolutionary divergence (Rokyta et al. 2015; Amazonas et al. 2018; Hofmann et al. 2018; Casewell et al. 2020; Colis-Torres et al. 2021). Additionally, snake venom systems are attractive models because of their direct relationships between venom gene expression, venom protein production, and venom phenotype (Casewell et al. 2012, 2013; Rokyta et al. 2015; Holding et al. 2016; Zancolli and Casewell 2020). Among snakes, the Prairie Rattlesnake (*Crotalus viridis*) has emerged as a model for studying among-population venom variation (Smith et al. 2023), and for understanding the glandular physiology and gene regulatory mechanisms associated with venom expression (Schield et al. 2019a; Perry et al. 2020, 2022; Westfall et al. 2023). Recent studies have identified candidate enhancers, promoters, TFs and TF binding sites (TFBSs) involved in venom gene regulation within this species (Perry et al. 2022) and have used single-cell approaches to confirm the roles of distinct TFs in regulating different venom loci (Westfall et al. 2023). These studies provide key foundations for exploring how differences in venom gene regulatory components may underlie the extensive variation in venom expression in *C*. *viridis* and related species. Notably, *C. viridis* venom phenotypes differ significantly in the primary components of their venom profile between southern and northern populations, with venom dominated by myotoxins in northern populations, versus snake venom metalloproteinases (SVMPs) in southern populations (Smith et al. 2023). Compared to *C. viridis*, closely related species (including *C. oreganus concolor, C. o. lutosus,* and *C. cerberus*) display remarkably different venom composition, including variation in expression levels of distinct venom families, as well as variation in certain paralogs within gene families (Mackessy 2010). Accordingly, this evolutionary variation provides a rich system to investigate the fundamental functional genomic underpinnings of venom phenotypic variation.

Here, we integrate multilevel functional genomic datasets and whole-genome resequencing data from Prairie Rattlesnakes (*C. viridis*) and three closely related species (*C*. *oreganus concolor, C*. *o*. *lutosus,* and *C*. *cerberus*) to survey the gene regulatory mechanisms underlying venom variation within this clade. Our sampling design is optimized to maximize phenotypic variation in venom composition across a continuum of genomic divergence in a relatively shallow phylogenetic transect of populations and species (<5 MY divergence), enhancing our ability to link changes in gene regulatory features to variation in phenotype. We use these data to explore variation in phenotype and regulatory features, such as trans-factor expression differences, chromatin and nucleotide differences at CREs, and evidence for differences in TF occupancy at CREs that exists within and between species.

We address the overarching hypothesis that that venom phenotypic variation is driven by underlying gene regulatory variation, including variable expression of relevant transcription factors, as well as chromatin state, TF occupancy, and nucleotide variation at CREs. We also hypothesize that diversity in a subset of gene regulatory network features might play consistent and dominant roles in driving expression variation, and that these patterns are consistent across all genes or paralogs within gene families. To test these hypotheses, we integrate mRNA-seq and proteomics to measure venom expression and composition, ATAC-seq data to compare chromatin accessibility and evidence of TF occupancy, and genome resequencing data to understand the contributions of CRE nucleotide differences among snake lineages and across venom genes and gene families. Our initial results indicated that the predictive importance of regulatory features is highly gene-specific. Based on this finding, we explore several gene-specific examples in detail, which individually highlight the diversity of distinct regulatory mechanisms (or combinations of mechanisms) that appear to impact gene expression differences.

## Results

### Variation in Venom mRNA and Protein Expression

**Fig. 1.**
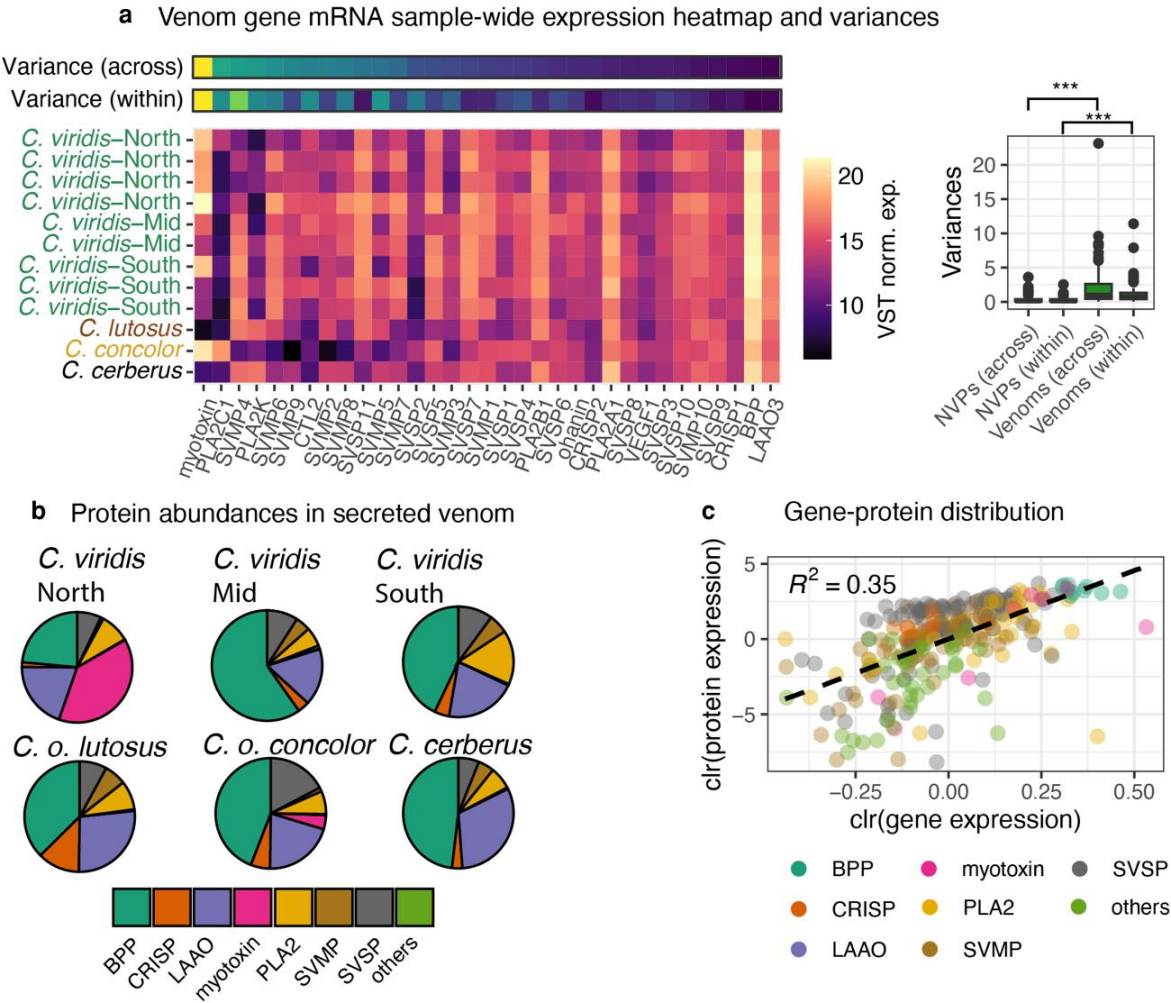
Toxin genes and their derived proteins display high expression variation. a) Venom gene expression for all individuals displayed as a heatmap. Variance, across all samples (across) and within *C. viridis* (within), in gene expression is shown as two rows above the heatmap, with brighter colors indicating higher variance and darker colors lower. Note that variances have been square root transformed to aid visualization; unscaled variances can be found in supplementary fig. S4, Supplementary Material online. To the right, the boxplot shows expression variance for venom genes and select nonvenom paralogs (a disintegrin and metalloproteinases (ADAMs), phospholipase A2s, beta-defensins and serine proteases; collectively NVPs; full list found in supplementary fig. S3, Supplementary Material online), both across all samples and within *C. viridis*. The asterisks represent *P*-values of a 2-sample t-test comparing groups (*** *P* < 0.01). Only significant comparisons are shown. b) Averaged venom protein abundances for each sampling group are displayed as pie charts. c) Linear correlation between protein and gene abundances. Gene and protein abundances were transformed using centered-log ratio (clr) transformation.

To quantify venom expression differences in an evolutionary context, we measured mRNA expression from both left and right venom glands of 12 individuals from four species and subspecies (supplementary table S1, Supplementary Material online). Venom genes exhibiting low expression across all samples were manually identified and subsequently excluded from all analyses (supplementary fig. S1, Supplementary Material online). We found no evidence of substantial differences in mRNA expression between left and right glands from the same individual, particularly for venom genes (supplementary fig. S2, Supplementary Material online). Therefore, we combined left and right gland expression data per individual to provide estimates of gene expression for most downstream analyses, unless otherwise noted. Our mRNA-seq data demonstrated substantial diversity in the gene expression of many venom genes across individuals, particularly myotoxin a/crotamine (hereafter myotoxin) and SVMPs (Fig. 1a). This variation is significantly greater than that observed in nonvenom paralogs (nonvenom metalloproteinases, phospholipase A2s, serine proteases, and beta-defensins; supplementary fig. S3, Supplementary Material online). Both within and across species, venom gene expression variation was the highest in myotoxin, a gene with a high degree of copy number variation (Gopalan et al. 2022) and several SVMP paralogs, the latter of which represent 9 out of the 20 most variably expressed venom genes across all samples, and 8 out of 20 within *C. viridis* (supplementary fig. S4, Supplementary Material online). This is consistent with prior evidence that proteomic variation in SVMP and myotoxin are major axes of venom variation across the range of *C. viridis* (Smith et al. 2023), which we find also applies to cross-species comparisons (e.g. *C. o. lutosus* expresses SVMPs relatively highly and myotoxin lowly, while *C. o. concolor* expresses the opposite profile). Other venom gene families with highly variable expression include phospholipases A_2_ (PLA2s) and snake venom serine proteases (SVSPs; supplementary fig. S4, Supplementary Material online).

Venom proteomic profiles were broadly consistent with venom toxin abundance inferred from mRNA-seq data (Fig. 1b), and a principal component analysis (PCA) of venom proteome composition across individuals separated species primarily by PC1 (73.12% variance explained), and populations of *C. viridis* by PC2 (12.02% variance explained; supplementary fig. S5, Supplementary Material online). To estimate the relationship between venom gland-derived venom gene mRNA expression and venom protein abundance, we followed the method of Rokyta et al. (2015) to scale and transform count-based gene expression (VST-normalized counts) and protein abundances (estimated from chromatographic peak intensity) using the centered-log ratio transform for each venom gene and its matched protein per individual (*R*^2^ = 0.35) (Fig. 1c).

### Venom-associated TF Expression Correlates With Venom Variation

**Fig. 2.**
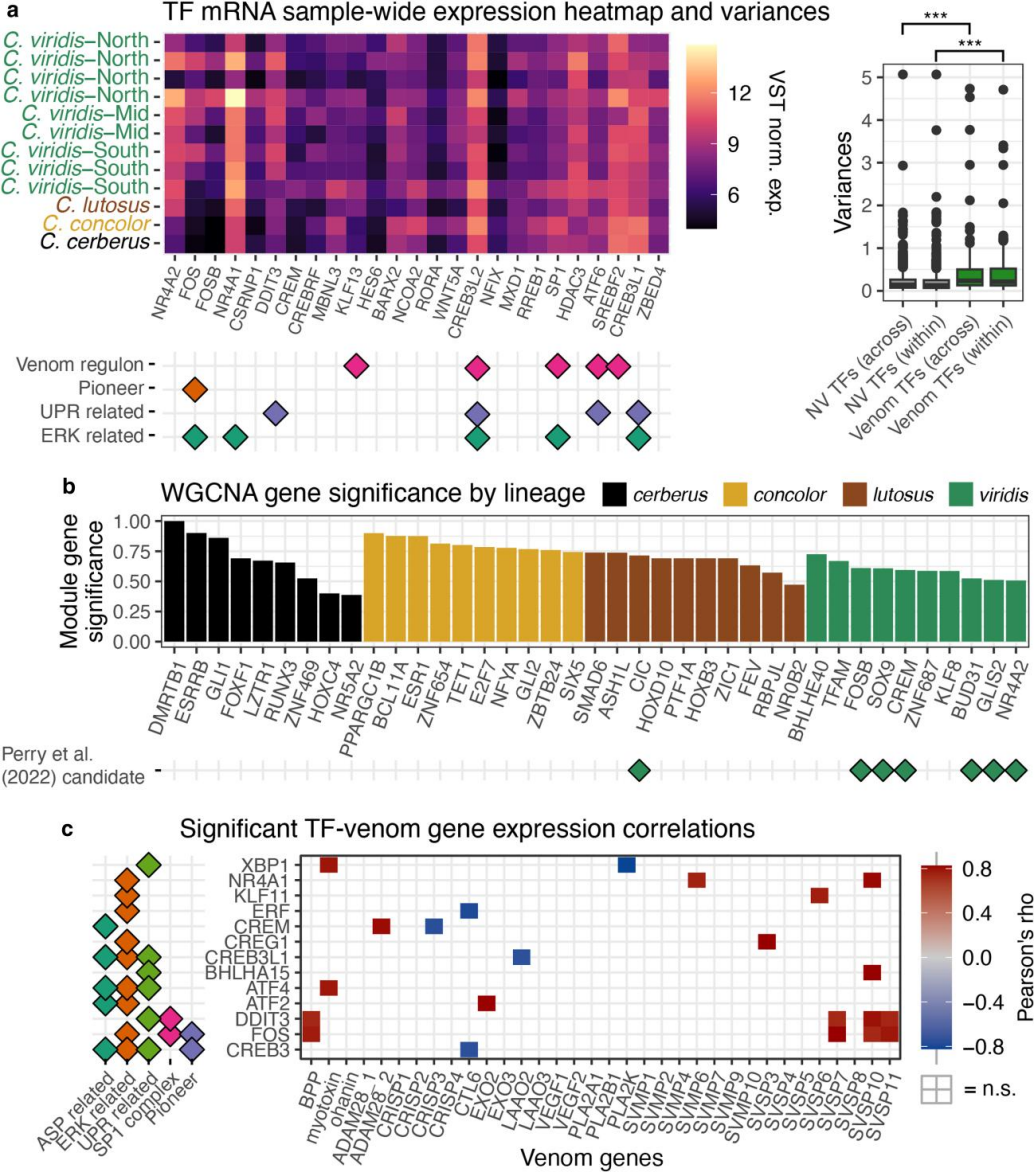
TF expression varies across lineages, suggesting role of trans-factor expression in venom variation. a) The top 25 TFs sorted by expression variance across all samples. To the right, the boxplot shows VST expression variances for all venom-associated TFs (from Perry et al. 2022; N = 161) and TFs not associated with venom, both within *C. viridis* and across all samples. The asterisks represent *P*-values of a 2-sample t-test comparing groups (***P < 0.01). b) WGCNA gene significance for TFs within the co-expression module that is most significant for each lineage variable. The functional annotations below 2a were taken from Perry et al. (2022) for pioneer TFs, UPR and ERK related TFs, and Westfall et al. (2023) for venom regulons. c) The matrix of Pearson's correlation coefficients between expression of candidate TFs and venom genes are displayed only for significant correlations. Pearson's rho scalebar on the right represents positive negative correlations. An uncolored box represents not significant (n.s.) correlations. Functional annotations come from Perry et al. (2022) and Westfall et al. (2023).

As an initial step to understand how differences in gene regulatory components explain venom gene expression, we focused on differences in trans-regulatory factor (TF) expression. We find that the top ranked TFs by expression are also often implicated in venom regulation (Perry et al. 2022; Westfall et al. 2023). TF expression varies considerably both within and among species, especially when compared to a background set of TFs not implicated in venom regulation (Fig. 2a). We also used DESeq2 (Love et al. 2014) to assess differential expression within and across species. However, we did not find evidence for differentially expressed TFs within different *C. viridis* populations. Across species, we did find evidence for the differential expression of 15 TFs: DLX3, EOMES, GABPA, GATA4, GATA6, HNF4A, MYF6, MYOG, NR1H4, PAX1, PBX1, SOX13, TBX19, TFAPC2, and VAX1, which, along with known TFs of importance (Perry et al. 2022) we include for downstream analyses of TF binding analysis.

This suggests that venom expression variation may be partly driven by differences in the expression of trans-regulatory factors, especially across species. To investigate this further, we tested for evidence of distinct co-expression modules between populations and species which may correlate with species identity by analyzing global venom gland mRNA data (including all genes) using WGCNA (Langfelder and Horvath 2008), through the estimation of module-gene significance values (Fig. 2b). Here, we analyzed left and right venom gland samples separately as biologically relevant replicates to increase power to detect co-expression modules. These modules were dominated by TFs, and modules with high scores in *C. viridis* include many TFs previously implicated in regulating *C. viridis* venom composition (Perry et al. 2022; supplementary table S2, Supplementary Material online). In addition to venom-associated TFs, the top *C. viridis* co-expression module also included venom genes (including CRISPs, SVSPs, SVMPs, and CTLs), chromatin regulators, and other TFs related to ERK and UPR signaling—key pathways hypothesized to coordinate venom expression (Perry et al. 2022; Westfall et al. 2023). We find each species is associated with distinct co-expression modules, indicating evolutionary lability in trans-acting factor expression (supplementary fig. S6, Supplementary Material online). Differences between genes comprising these species-specific modules include venom-associated TFs (Perry et al. 2022), as well as TFs without prior known links to venom regulation (Fig. 2b).

To test for evidence that the expression of TFs was predictive of venom expression, we calculated gene–gene expression correlation coefficients between venom-associated TFs and venom gene expression across all samples and find several TFs whose expression is highly predictive of the expression of specific venom genes (Fig. 2c). For example, expression of myotoxin is strongly correlated with the expression of XBP1 (ρ = 0.80; *P*-value = 0.001) and ATF4 (ρ = 0.79; *P*-value = 0.002), the latter of which has been previously predicted to have a binding site in the myotoxin promoter (Gopalan et al. 2022). Additionally, FOS and DDIT3 are significantly (*P*-value < 0.05) positively correlated with the expression of four distinct venom genes: BPP, SVSP7, SVSP10, and SVSP11 (ρ = 0.75 to 0.82).

### Broad Evidence that CRE Chromatin State, SNPs and TF Binding Underlie Venom Variation

**Fig. 3.**
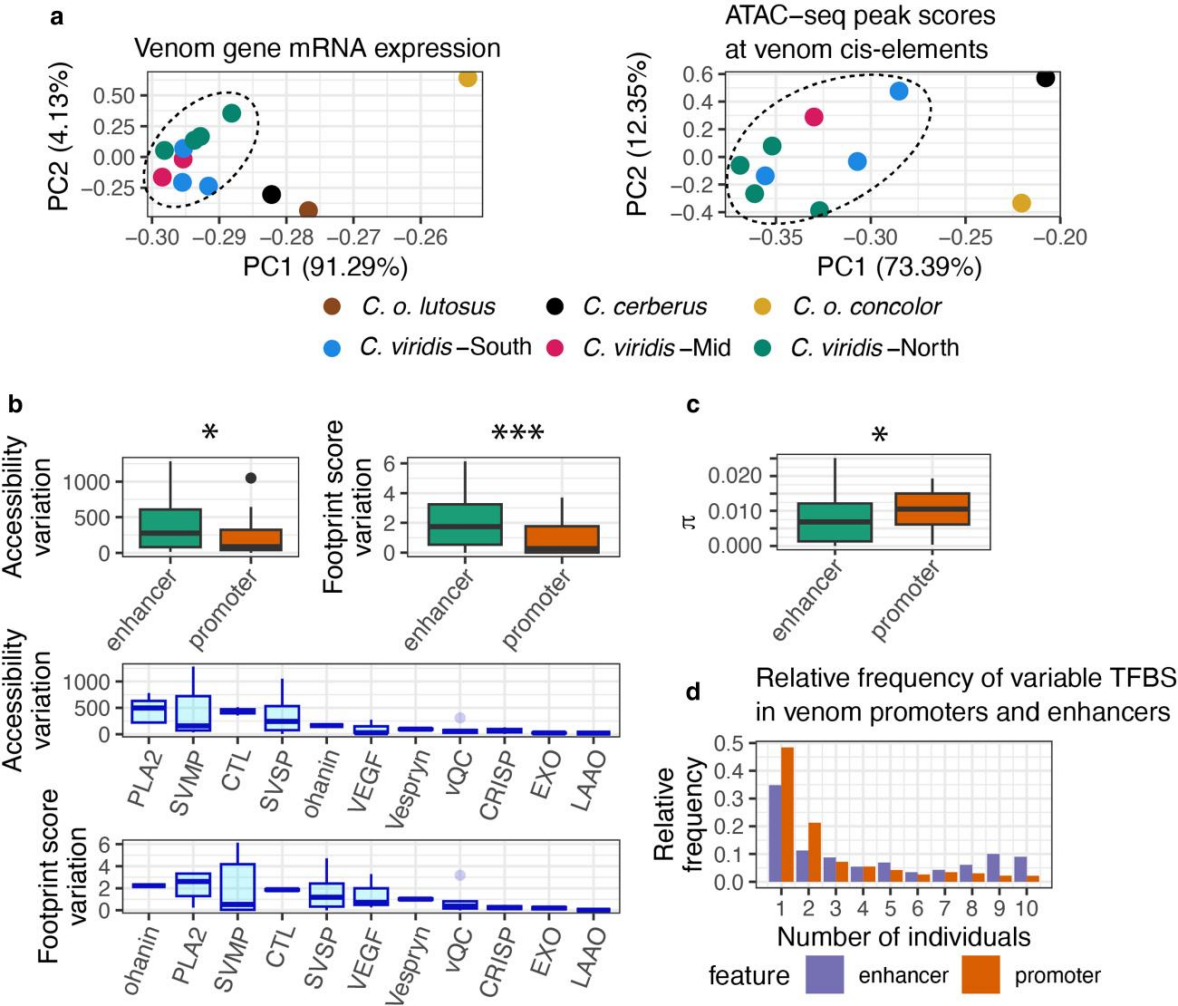
Abundant chromatin accessibility, TF binding and standing nucleotide variation exist in venom CREs. a) PCAs of venom gene mRNA expression and ATAC-seq peak scores at venom cis-elements (promoters and enhancers of venom genes) demonstrate variance partitioning across populations and species, corresponding to the two main PC axes. The dashed line encircles *C. viridis* samples. b) Chromatin accessibility and peak accessibility variation for enhancers and promoters. The variance sorted accessibility and footprint scores across venom gene families are shown below. c) Nucleotide diversity (π) for venom enhancers and promoters. d) Frequency of variable TFBSs within enhancers and promoters across samples. This is interpreted in a similar manner to a site frequency spectrum, where each bar represents the fraction of TFBSs that are shared by that many individuals. Asterisks above boxplots indicate statistical significance for parametric 2-sample t-tests: *P < 0.05; ***P < 0.001.

In a prior study, we integrated ChIP-seq, ATAC-seq, and Hi-C data to infer CREs associated with venom loci in *C. viridis* (Perry et al. 2022), which we use here as a base set of known CREs for downstream analyses. To investigate the roles of cis-regulatory feature variation, we first characterized differences in ATAC-seq derived chromatin accessibility, ATAC-seq derived TF footprint score (likelihood of TF occupancy) within venom gene CREs, and genotype derived nucleotide variation at venom gene CREs. The similarity in the PCAs of chromatin accessibility at venom gene CREs and of venom gene expression suggests that these two metrics broadly covary according to population ancestry (Fig. 3a). To further dissect the relevance of specific types of CRE variation, we quantified variation in CRE accessibility and footprint scores at promoters and enhancers and find that enhancers consistently showed greater variation in both accessibility and TF binding compared to promoters (Fig. 3b). We also find that the CREs of venom gene families that show the greatest accessibility and footprint score variation are those that displayed the highest and most variable expression, including PLA_2_s, SVMPs, SVSPs, and CTL2, pointing to a high-level correspondence between mRNA-seq and ATAC-seq data (Fig. 3b; supplementary fig. S4, Supplementary Material online). Despite this, we do not find statistical evidence that variation in accessibility or in footprint scores are linearly correlated with variation in gene expression, suggesting multifactor, and nonlinear interactions play a larger role.

Because nucleotide variation at CREs can impact TF binding and thus gene regulation, we also assessed nucleotide diversity (π) from sample-wide single-nucleotide polymorphisms (SNPs) detected at venom gene CREs (Fig. 3c). This subset of SNP calls were of high quality, with an average cross-sample depth of 54.2, and average VCF quality score of 81 (probability of base call error ≈ 1/10^8^ on average; supplementary table S3, Supplementary Material online). We find that promoter sequences tended to be more variable than enhancers, despite enhancers having greater variation in chromatin accessibility and TF occupancy (Fig. 3b). Of 41 predicted venom enhancers and 50 venom promoters, only 7 enhancers showed no genetic variation (π = 0) across all individuals, 3 of which were SVSP enhancers (supplementary fig. S7, Supplementary Material online). To assess the functional implications in cases where we detected genetic variation, we predicted variants in CREs that modified the presence or the absence of TFBS and used this to assess the frequencies of these variants across individuals (Fig. 3d). We find that while nucleotide variation affecting TFBSs is common (CRE variants affect the presence and absence of 9395 TFBSs at venom gene CREs), 47% of variable TFBSs at promoters and enhancers are unique to a single sampled individual, highlighting the extensive variation in CRE sequences that exists across populations and species that is likely relevant to variation in venom expression.

### Distinct Types of Regulatory Feature Variation Explain Expression of Distinct Venom Genes

**Fig. 4.**
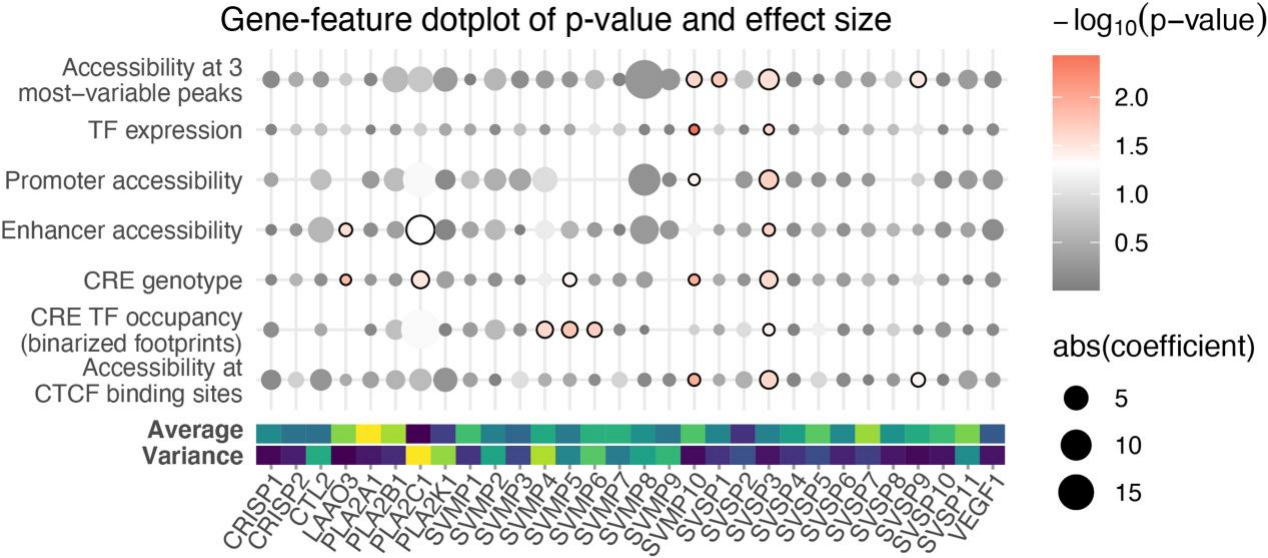
Linking toxin gene expression and regulatory variation. Results of the linear modeling on a gene-by-gene basis. Absolute values of regression coefficients and log-transformed *P*-values from multiple linear regression are shown as point size and point color respectively. Absolute values were used to assess only the effect size of the inputs. The color scale shifts from gray to red at the point of significance (*P* < 0.05). Points where the correlation is significant are also outlined in black. Sample-wide average gene expression and gene expression variance are shown as colored bars below, where brighter colors indicate higher values.

Considering the diversity and high dimensionality of regulatory features that may affect gene expression, we first used phylogenetic PCA to reduce dimensionality of regulatory feature variation, then applied multiple linear regression using these principal components as predictor variables to identify what types of regulatory variation are associated with gene expression variation at a broad scale. These features include accessibility, both genotype and TF occupancy at previously identified CREs (Perry et al. 2022), expression of venom-regulating TFs, and accessibility at other potential cis-elements such as variably accessible peaks and binding sites of the insulator protein CTCF across venom gene clusters. Quantifying accessibility at CTCF loci is important in understanding potential variation in the structure of topologically associated domains, which can cause expression differences between physically adjacent venom genes (Perry et al. 2022). As a prerequisite for modeling, we ensured that candidate genes had a well-understood genomic context (i.e. sequencing of the adjacent region in the reference, enhancer predictions and CTCF predictions). This precluded a focus on myotoxin or BPP, which have genomic contexts that have yet to be well resolved. We explored relationships between regulatory variation and venom expression on a gene-by-gene basis (Fig. 4). We find that the most predictive regulatory characteristics are highly gene-specific, although CRE genotype, TF occupancy at previously identified venom gene CREs, as well as de novo identified (previously unannotated) ATAC-seq peaks predict expression for most venom genes. TF occupancy of venom-regulating TFs for example correlates with the expression of three physically adjacent SVMPs paralogs (SVMP4, SVMP5, and SVMP6). Some genes, such as LAAO3 and SVSP9, correlate with only a few specific regulatory feature types, while other genes (e.g. SVMP10 and SVSP3) respond to a suite of features. Nonsignificant model results do not seem to be related to low gene expression in most cases, though high feature coefficients appear to be the result of high expression variance in at least the case of PLA2C1. Overall, our linear models highlighted a subset of venom loci for which gene expression was strongly associated with variation in regulatory factors, and in some cases, with gene-level specificity. The results of the linear modeling provided a set of potential genes of interest with respect to understanding the effects of specific regulatory inputs, and so we further investigated several specific venom loci in gene-specific vignettes.

### SVMP6 Expression Responds to Variable Trans-factor Binding at its Enhancer

**Fig. 5.**
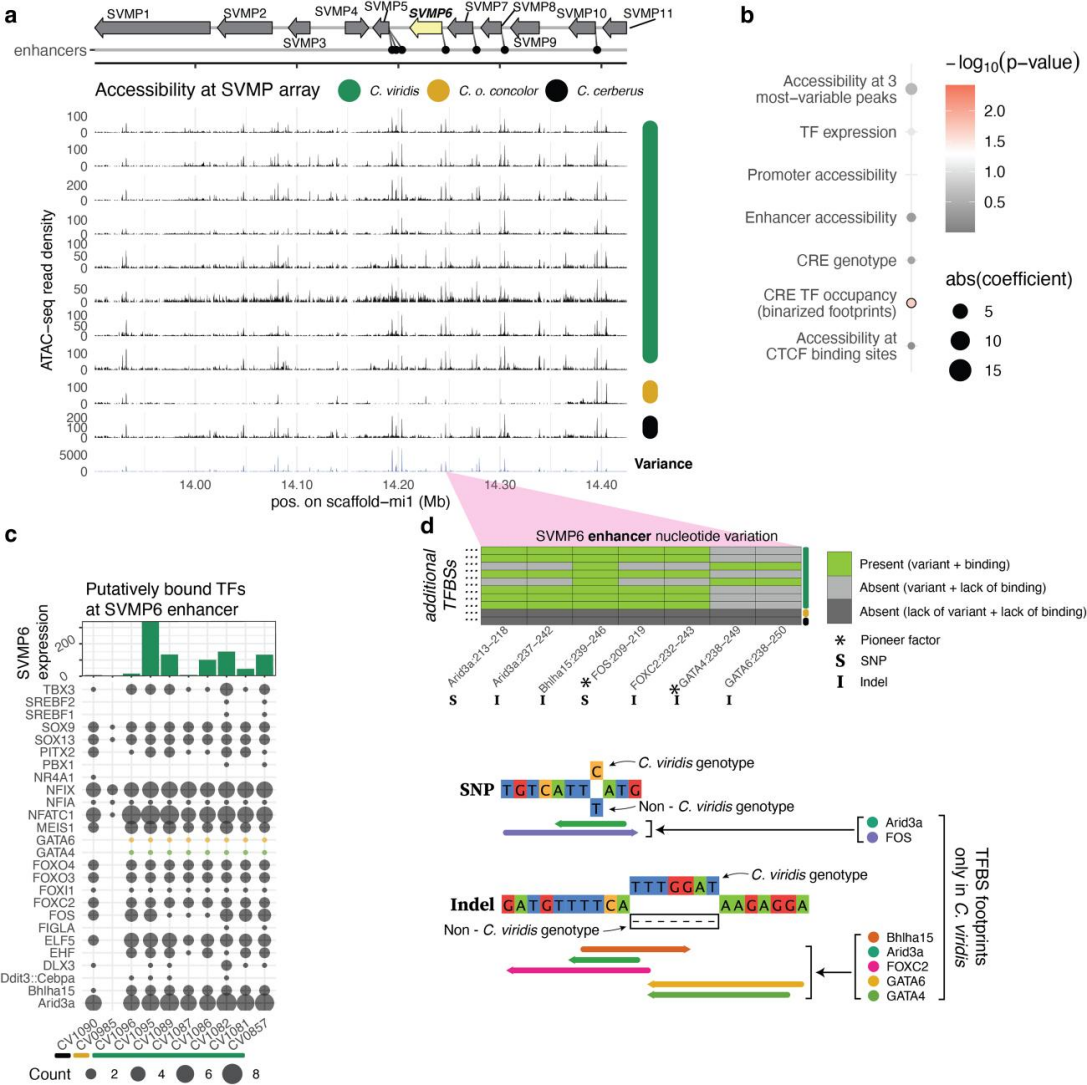
Nucleotide variation causes TF occupancy differences at enhancer, driving SVMP6 expression variation. a) The SVMP gene array and enhancers redrawn from Perry et al. (2022). Venom gland ATAC-seq for *C. viridis* and non-*C. viridis* individuals are shown as read pileup tracks. Variance in ATAC-seq density is shown as the bottom-most track. b) Results of multiple linear modeling for SVMP6 redrawn from Fig. 4. The significant feature is circled in black. c) TF binding frequency at the SVMP6 enhancer is shown, with SVMP6 expression displayed as a histogram at the top. GATA4 and GATA6 are highlighted with different colors. The expression is shown as DESeq2-normalized counts in thousands. d) The SNP and indel variants that modify the TF occupancy at TFBS sequences is shown for TFBS sequences in the SVMP6 enhancer. Below this, TFBS motifs which are affected by the SNP and indel variants are drawn onto the sequence, as well as the genotypes of *C. viridis* and non-*C. viridis* individuals. The direction of each motif is indicated by the arrow, and individual colors represent separate TFBSs.

Considering that SVMP gene and proteomic expression is highly variable within and between species (Fig. 1a, and Smith et al. 2023), we compared chromatin accessibility across samples at the SVMP gene cluster and find that variance in accessibility tends to be much higher at enhancers than promoters (Figs. 3b and 5a; supplementary fig. S8, Supplementary Material online). To investigate these relationships further, we focused on the SVMP paralog SVMP6, which showed highly variable gene expression (Fig. 1a, supplementary fig. S4, Supplementary Material online), high levels of nucleotide diversity at its enhancer (supplementary fig. S7, Supplementary Material online), and significant correlations between SVMP6 expression and TF occupancy at CREs based on linear modeling (Fig. 5b). None of these patterns are confounded by excessive structural variation at the enhancer (supplementary fig. S9, Supplementary Material online). We first assessed TFBS occupancy differences (based on ATAC-seq footprint scores) between samples at the SVMP6 enhancer by quantifying the total number of binding events for each TF and find evidence for variable TFBS occupancy across samples of *C. viridis*, and very low predicted levels of TFBS occupancy in *C. o. concolor* that corresponds with very low SVMP6 expression in this species (Fig. 5c). The high degree of variation in TFBS occupancy suggest that there may be differences in cell populations with respect to TF binding, or that TFs may cooperatively bind to activate the enhancer. ATAC-seq footprint scores suggest that TFs such as GATA6 and GATA4 are bound only in *C. viridis*, while others such as PITX2, EHF and DDIT3 vary both in frequency of binding and presence across samples. This indicates that variation in TF binding at the SVMP6 enhancer is indeed associated with variation in gene expression across samples within and among species.

To investigate how evidence for variable TF binding at this enhancer may be related to the nucleotide variation at this locus, we focused on enhancer variants at known TFBSs that were also associated with differences in estimated TF occupancy across samples. This highlighted two variants, one SNP and one indel, which together impact TFBSs of as many as six TFs in the *C. viridis* samples and are absent in *C. o. concolor* and *C. cerberus* (Fig. 5d). These differentially bound TFs include two pioneer transcription factors (GATA4 and FOS) that can initiate regulatory events by opening chromatin (Cirillo et al. 2002; Fleming et al. 2013). This example highlights the roles of TF occupancy differences, which can be driven by allelic variants at enhancers, as a mechanism leading to differential gene expression within and between species.

### SVSP9 Expression Responds to Accessibility at Enhancers, Silencers, and Insulation by CTCF

**Fig. 6.**
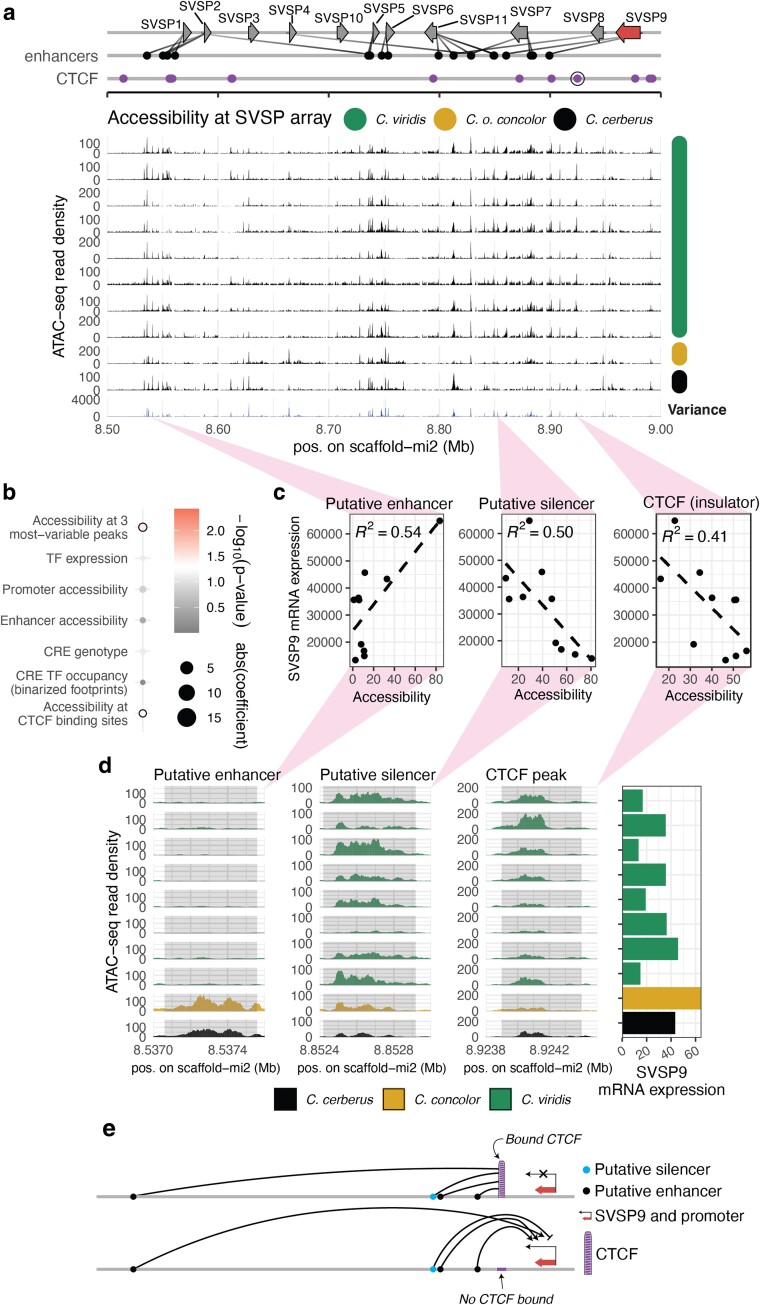
*De-novo* and CTCF-bound loci explain SVSP9 expression. a) The SVSP gene array and its predicted enhancers (redrawn from Perry et al. 2022), with CTCF binding inferred from ChIP-seq (Perry et al. 2022) shown below. The locus with accessibility that was significantly correlated with SVSP9 expression is circled. Venom gland ATAC-seq for *C. viridis* and non-*C. viridis* individuals are shown as read pileup tracks. Variance in read density is shown as the bottom-most track. b) Results of linear modeling for SVSP9, redrawn from Fig. 4b. The significant features are outlined with a black circle. c) Linear regressions between chromatin accessibility at the three loci of interest (a putative enhancer, silencer and a CTCF-bound locus) and SVSP9 gene expression. All linear models are significant at *P* < 0.05. d) Accessibility landscapes at the loci of interest are shown with a SVSP9 gene expression histogram shown at the far right. The light gray rectangles show the location of ATAC-seq peaks called by MACS2. Peaks have been centered and length standardized. e) A proposed model for how SVSP9 gene regulation responds to various input loci, and how this may be inhibited by CTCF binding.

SVSPs represent a major component of rattlesnake venoms and show high degrees of gene expression variation and ATAC-seq variation across our samples compared to other venom genes (Figs. 1a and 3b, and 6a). For one SVSP paralog, SVSP9, our linear modeling suggests its expression is significantly correlated with accessibility at a known CTCF-binding site, and accessibility at additional nonannotated loci (loci with highly variable accessibility not previously identified as a CREs; Fig. 6b). To further investigate these loci, we examined ATAC-seq density across samples at the entire SVSP locus (Fig. 6a), and at the three ATAC-seq peaks within this gene cluster that showed significant (*P* < 0.05) correlations between their accessibility and SVSP9 gene expression (Fig. 6c). For both regions not previously annotated, we find moderately strong individual correlations (*R*^2^ > 0.5) between their accessibility and SVSP9 expression, but with opposing effects, suggesting one may represent a putative enhancer while the second may represent a putative silencer (Figs. 6c and d). We also find evidence that accessibility at a previously predicted binding site for the insulator protein CTCF (Perry et al. 2022), located between the promoter of SVSP9 and its putative enhancers, is negatively correlated with SVSP9 expression (Figs. 6a c, and d). These findings provide evidence for how gene expression may vary across populations and species through the modulation of chromatin accessibility at CREs through both positive (enhancer) and negative (silencer and CTCF) gene regulatory interactions. Notably, these findings also highlight the potential role of the insulator protein CTCF, through its regulation of chromatin loops and enhancer–promoter interaction, in generating inter-population and inter-species gene expression diversity (Fig. 6e).

### Variation in Myotoxin Expression is Predicted by TF Binding and Expression

Though the genomic context of myotoxin remains poorly resolved, which has prevented identification of distal regulatory loci, it is notable for being the most variably expressed venom gene across our sampling (Fig. 1a). Our transcriptomic data identified strong correlations between expression of myotoxin and two TFs, ATF4, and XBP1 (Fig. 2c). The promoter sequence is known and is completely conserved across sampled individuals (supplementary fig. S7, Supplementary Material online), which, based on ATAC-seq derived TF footprint scores, does not contribute strongly to TF binding differences (supplementary fig. S10, Supplementary Material online). The promoter is predicted to be bound by ATF4 in all samples with accessible chromatin, and promoter accessibility corresponds with gene expression (supplementary fig. S10, Supplementary Material online). While no evidence of XBP1 binding was detected in the promoter, it is possible that it may bind an enhancer that has yet to be identified, form a complex with other TFs and thus not leave detectable chromatin footprints, or play a role in higher-level regulation of ATF4 or other myotoxin-regulating factors.

### SVSP2 Expression Knocked out by Individual-specific Enhancer Deletions

In contrast to the CREs of other venom gene families, SVSP enhancers generally have very little or no nucleotide diversity (supplementary fig. S7, Supplementary Material online). Although our linear modeling provided no clear evidence of strongly associated genomic features, we find that other non-modeled features (e.g. structural variants) may be relevant (supplementary figs. S11 and S12, Supplementary Material online). The SVSP2 locus stood out as it was among the most variably expressed venom genes in *C. viridis* (supplementary fig. S4, Supplementary Material online), yet its two adjacent enhancers (PER17 and PER 17) showed no SNP variation across samples (supplementary fig. S7, Supplementary Material online). To test for potential effects of larger structural variation, we analyzed genome resequencing read density for *C. viridis* individuals versus the reference genome and find evidence for a several kilobase deletion affecting these enhancers in two *C. viridis* individuals from southern latitude populations (supplementary fig. S12, Supplementary Material online), which corresponds with low expression of this gene in these individuals (supplementary fig. S11, Supplementary Material online). These results highlight a case where gene expression variation may occur through the action of larger effect structural variation that exists among populations within species.

## Discussion

While the rapid evolution of GRNs and the subsequent changes in gene expression are likely major drivers of adaptation and functional divergence (Wittkopp 2007; Emerson and Li 2010; Thompson et al. 2015), identifying the relative contributions of distinct regulatory components to gene and gene family expression variation, and ultimately phenotypic variation, remains challenging (Romero et al. 2012). Snake venom systems provide a uniquely powerful system, with extensive variation in venom gene expression in multiple gene families across closely related populations and species, to identify how variation in gene regulatory components contributes to gene expression variation. The ability to simultaneously measure matched protein, mRNA, and regulatory variation from the same individual during venom production affords the opportunity to more clearly link relationships between phenotype, gene expression, and regulatory variation in a comparative experimental framework. We leveraged this system here to highlight remarkable fine-scale evolutionary variation underlying phenotypic variation in a keystone adaptive trait (venom), and to further link specific mechanisms of regulatory variation to phenotypic variation.

We find that chromatin accessibility at CREs, CRE genotype variation, and predicted TF binding all influence gene expression, but to varying degrees across specific genes and gene families. Much of this is driven by high levels of nucleotide and accessibility variation at venom gene CREs, both between and even within species. We also find evidence that the specific types of gene regulatory components that contribute to venom expression variation are not only diverse but are also remarkably gene and gene family specific. In addition to canonical expectations that chromatin, TF-CRE interactions, and CRE genotype underly phenotypic variation, we also find evidence that trans-regulatory factor (i.e. TF) variation and variation in the action of the insulator protein CTCF may also play major roles in generating within and between species expression variation. Broadly, these findings establish expectations that even at shallow levels of divergence, a diversity of regulatory mechanisms may shape phenotypic variation, and that distinct genomic mechanisms may often dominate the modulation of gene expression for particular genes and gene families.

### Roles of Nucleotide, Chromatin Accessibility and TF Variation

Considering the fine scale of evolutionary divergence surveyed here, we observed notably high degrees of nucleotide diversity at venom gene CREs that provide substantial “raw material” for generating variation in TF binding and chromatin accessibility that may impact venom gene expression. Indeed, we find evidence that venom gene expression is frequently related to CRE chromatin accessibility as well as CRE genotype at these venom loci, likely because both factors are key determinants of TF occupancy (Wittkopp and Kalay 2012). We showcase the regulation of the venom metalloproteinase SVMP6 to demonstrate how mutations influencing expression can confer species-specific TF binding, a pattern supported by linear modeling of regulatory network effects.

TF binding and regulatory activity can also vary depending on the expression of the transcription factors themselves. Our results suggest different suites of co-expressed TFs, many of which have been previously implicated in venom regulation, follow population- and species-specific trends, implying that distinct venom-regulating TF expression also contributes to venom gene expression variation. Based on gene expression correlations, some TFs, such as the pioneer factor FOS (Fleming et al. 2013) and DDIT3 appear to co-regulate venom genes. Both TFs are components of the AP-1 TF complex, a major regulatory complex stimulated by venom depletion (Luna et al. 2009) and are known to physically interact (Oughtred et al. 2021). Additional correlation-based evidence comes from the myotoxin gene, which correlates with the expression of ATF4 and XBP1. These findings are notable because they highlight the correspondence between inferences from transcriptomic correlations and independent inferences of TF-CRE interactions from ATAC-seq data, both of which are consistent with prior inferences for the roles of the unfolded protein response (UPR) pathway, of which ATF4 and XPB1 are both members, in regulating venom genes. Being a gene of interest based on expression variation, myotoxin differs from most other venom gene families, such as SVMPs, SVSPs, and PLA2s, in that the genome assembly and annotation of this region remains poorly resolved (Schield et al. 2019a; Gopalan et al. 2022). From what we do understand, myotoxin paralog number appears to vary substantially even within *C. viridis*, yet paralogs appear to be identical in protein-coding sequence (Gopalan et al. 2022). Thus, unlike other multigene venom families, myotoxin expression may be primarily modulated by dosage (e.g. gene copy number variation), although our data also suggest that regulation of trans-acting factors (and potentially chromatin variation) may also play key roles.

Taken together, our results suggest that most phenotypic differences in venom between species are likely driven by changes in TF expression in the venom gland, whereas expression is tuned at finer scales by functional nucleotide variation and variable chromatin access at CREs. Indeed, recent findings have supported the hypothesis that the larger effect-size changes of the trans-regulatory environment may tend to evolutionarily persist when restricted to only some tissues (Barr et al. 2023). This would suggest that a fraction of observed venom compositional variation between lineages may result from divergence in trans-regulatory factor expression variation in the venom gland.

### A Role for Variation in CTCF-mediated Insulation in Expression Variation

The protein CTCF, originally identified as a transcriptional repressor, is known to play broad roles as an “insulator” through its roles in defining chromatin boundaries and directing of chromatin looping structures that can modulate enhancer–promoter interactions (Lobanenkov et al. 1990; Ong and Corces 2014; Ren et al. 2017). Prior studies on snake venom regulation have identified the roles of CTCF in directing gene regulatory interactions across multiple venom gene clusters (Schield et al. 2019a; Liao et al. 2021; Perry et al. 2022). Based on modeling and additional analyses, we find evidence for the effects of binding of the insulator protein CTCF on gene expression variation. Our results suggest that CTCF-mediated insulation may be used to direct gene expression changes across recent evolutionary scales. The example demonstrating this leverages the complex regulatory architecture of the viperid SVSP cluster, which is a result of chromatin loops, often guided by CTCF, forming topologically associated domains isolating paralogs from their neighbors (Perry et al. 2022). Our sampling encompassing fine-scaled evolutionary variation has allowed us to identify additional features and associations related to the regulatory nature of SVSP9. We find that accessibility at a known CTCF-bound locus between the promoter and enhancers of SVSP9 produces a negative correlation with gene expression, consistent with the expected effects of CTCF as an insulator that can negatively mediate enhancer–promoter interactions through its action in mediating chromatin loops. While we do identify two new putative regulatory loci and a regulatory role of a CTCF locus for SVSP9, given the often multienhancer nature of viperid venom genes (Perry et al. 2022), this does not exclude the presence of other distal regulatory loci beyond our search space which could more accurately explain the regulatory nature of SVSP9.

### Roles of Functional and Structural Variation at CREs

Our findings also provide new insight into the potentially distinct roles and mechanisms of functional diversity in promoters and enhancers in the context of evolutionary modulation of gene expression, with enhancer variation being dominated by chromatin variation while promoter variation is dominated by genotype variation. In snake venom genes, enhancers tend to be less genetically variable than promoters, yet show higher variation in chromatin accessibility and TF binding. Whether this is a generalizable trend, or specific to venom genes, remains unresolved. A recent study has suggested that snake venom genes may have elevated allelic diversity due to pervasive balancing selection (Schield et al. 2022), which may also drive elevated diversity at the proximal promoter loci of these genes but be reduced as more distant enhancer loci.

Prior studies on snake venom gene clusters have linked venom composition and gene expression variation to larger-scale genomic mechanisms such as structural diversity, which drives venom compositional variation between species (Casewell et al. 2011; Dowell et al. 2016; Giorgianni et al. 2020; Margres et al. 2021). Additional studies have also quantified chromatin accessibility (Margres et al. 2021; Perry et al. 2022) and DNA methylation (Margres et al. 2021), linking these to variation in expression across venom genes within single individual snakes (Margres et al. 2021; Perry et al. 2022). The work presented here extends the findings of prior studies through the integration of functional genomic data across multiple individuals and species that enables the contextualization of the evolutionary roles of chromatin state as well as genomic variation in generating venom gene expression. This now allows for a far more comprehensive understanding of precise mechanisms by which modifications to chromatin access and nucleotides at CREs act as regulatory inputs to tune a highly selected phenotype within and across species.

Several prior studies have attempted to define expectations for the roles of general cis- and trans-effects in driving divergent inter-species diversity through independently measuring cis-element activity, chromatin accessibility, and gene expression across species (Berthelot et al. 2018; Pizzollo et al. 2018; Edsall et al. 2019; Barr et al. 2023). Developing an integrated quantitative understanding of gene expression variation in the context of multiple forms of regulatory variation has, however, remained a challenge. The distinct nature of our experimental design here, using shallow-divergence comparative studies, holds great potential as an alternative and productive way forward for detecting molecular variation and linking these diverse sources of variation to their relevance in directing gene expression, particularly in model systems in which mutagenesis is not feasible.

One critical axis of gene regulatory variation that was not directly explored in this study is the role of noncoding RNAs. Prior studies have implicated miRNAs as key underlying factors that explain divergent venom expression patterns (Durban et al. 2013; Zheng et al. 2023), and long noncoding RNAs that may also be involved in snake venom diversity and regulation (Gopalan et al. 2022; Zheng et al. 2023) have been identified. Though one study alternatively found that posttranscriptional mechanisms play a negligible role in venom regulation (Rokyta et al. 2015), our comparison of mRNA versus protein abundance highlights multiple venom genes that show lower than expected protein abundance compared to mRNA abundance (including myotoxin, some PLA_2_s, and SVMPs), consistent with miRNAs playing a posttranscriptional regulatory role in venom composition variation. Future work to integrate the roles of noncoding RNAs more directly in modulating venom gene expression phenotypes would provide a more comprehensive, and likely more complex, understanding of the factors that ultimately modulate venom expression phenotypes and venom composition.

## Conclusion

Recent studies have used hybrid or cybrid experimental designs to provide valuable insight into the relative roles of cis- versus trans-gene regulatory components in modulating gene expression phenotypes. In contrast, this study represents one of a few (Wittkopp et al. 2008; Jones et al. 2012) that has interrogated naturally existing variation in GRNs at fine evolutionary scales. Consequently, it provides valuable baseline expectations for the extent and functional impacts of naturally occurring gene regulatory variants. Our findings highlight a surprisingly high degree of naturally occurring gene regulatory variation and the extensive diversity of underlying mechanisms that appear to play dominant roles in different genes and gene families. This relatively small-scale study suggests that more powerful larger-scale comparative functional genomics studies hold exciting promise as hypothesis-generating and testing platforms for gene regulatory function, and for inferring how regulatory variation may manifest in phenotypic variation.

## Materials and Methods

### Tissue Sampling

All animal collection, housing, and sampling was conducted according to an approved and registered IACUC protocol (2303D-SM-S-26; S.P. Mackessy) at the University of Northern Colorado, and animals were collected under approved state permits (Arizona, Colorado, Utah, New Mexico, and Texas). To initiate venom production, venom was manually extracted from both venom glands one day prior to sacrifice. Animals were anesthetized using isoflurane and humanely sacrificed by severing the spinal cord. Left and right venom gland, right accessory venom gland, skin, pancreas, skeletal muscle, heart, and liver tissues were immediately dissected out and snap frozen in liquid nitrogen. For this study, only venom, blood, left and right venom gland tissues were used.

### mRNA-seq and Venom Protein Data Generation and Analysis

Total RNA was extracted from snap-frozen tissues using TRIzol reagent (Invitrogen Life Technologies, No. 15596026). For this study, all RNA extractions were performed in a single batch. A single left venom gland sample was excluded from the study due to poor data quality, leaving a total of 23 venom gland tissues. Library preparation and sequencing were performed by Novogene (Sacramento, California). Briefly, mRNA was selected from total RNA using poly-T oligo-attached magnetic beads, followed by fragmentation, reverse transcription, adapter ligation, and amplification by PCR. The library was quality checked for size distribution using a Bioanalyzer (Agilent 5400). mRNA libraries were then sequenced on an Illumina NovaSeq 6000 using 150 bp paired-end reads. Raw reads were quality trimmed using Trimmomatic v0.39 with the settings LEADING:20 TRAILING:20 MINLEN:32 AVGQUAL:30 (Bolger et al. 2014), and resulting paired reads were mapped to the annotated *Crotalus viridis* reference genome (NCBI GCA_003400415.2, Schield et al. 2019a) using STAR v2.7.9a (Dobin et al. 2013). Reads mapped to genic features in the reference annotation were counted by exon and summarized by gene using featureCounts v1.6.3 (Liao et al. 2014) to provide estimates of gene expression. Differential gene expression between *C. viridis* and non-*C. viridis* individuals, and individuals within *C. viridis* populations was performed using DEseq2 v1.30.1 (Love et al. 2014) in R (R Core Team 2022). TFs found to be differentially expressed across species were considered “of significance” and were appended to a previously generated set of TFs from a prior study (Perry et al. 2022) for the purposes of TFBS scanning (see below). DESeq2 was then used to produce library-size normalized count matrices (using the “counts’ command) and variance stabilizing transformed count matrices (using the “vst” command), the latter of which was used to produce heatmaps in R.

WGCNA (Langfelder and Horvath 2008) was used to perform module co-expression analyses and to estimate module-trait significance values. WGCNA was run twice with standard settings. It was run initially with all left and right venom gland samples (N = 23) to estimate module-trait significance values for species identity (i.e. *C. viridis*, *C. o. lutosus*, *C. o. concolor* and *C. cerberus*). To generate gene–gene correlation matrices from gene expression, Pearson's rho was calculated in R using the “rcorr” function from the “Hmisc” package (cran.r-project.org/web/packages/Hmisc) and the coefficient matrix was filtered for *P*-value < 0.05 and FDR < 0.1 to produce a significance-filtered TF-venom gene correlation matrix.

### Venom Proteomics

Lyophilized venoms were resuspended in 8 M urea/0.1 M Tris (pH 8.5), reduced with 5 mM TCEP (tris (2-carboxyethyl) phosphine) for 20 min, and alkylated with 50 mM 2-chloroacetamide for 15 min in the dark all at room temperature. Samples were diluted 4 times with 100 mM Tris–HCl (pH 8.5) and trypsin digested at an enzyme/substrate ratio of 1:20 overnight at 37°C. Digestion was stopped with formic acid (FA), and proteolytic peptides were purified with Pierce C18 Spin Tips (ThermoFisher Scientific). Samples were dried in a speed vacuum and resuspended in 0.1% FA.

Liquid chromatography-tandem mass spectrometry (LC-MS/MS) was performed using an Easy nLC 1000 instrument coupled with a Q Exactive HF Mass Spectrometer (both from ThermoFisher Scientific). Digested peptides were loaded on a C_18_ column (100 μM inner diameter × 20 cm) packed in-house with 2.7 μm Cortecs C18 resin, and separated at a flow rate of 0.4 μl/min with solution A (0.1% FA) and solution B (0.1% FA in acetonitrile) under the following conditions: isocratic at 4% B for 3 min, followed by 4% to 32% B for 102 min, 32% to 55% B for 5 min, 55% to 95% B for 1 min and isocratic at 95% B for 9 min. Mass spectrometry was performed in data-dependent acquisition mode. Full MS scans were obtained from *m/z* 300 to 1800 at a resolution of 660,000, an automatic gain control (AGC) target of 1 × 10^6^, and a maximum injection time (IT) of 50 ms. The top 15 most abundant precursors with an intensity threshold of 9.1 × 10^3^ were selected for MS/MS acquisition at a 15,000 resolution, 1 × 10^5^ AGC, and a maximal IT of 110 ms. The isolation window was set to 2.0 m*/z* and ions were fragmented at a normalized collision energy of 30. Dynamic exclusion was set to 20 s.

Fragmentation spectra were interpreted against a database containing translated sequences derived from a public transcriptome (Schield et al. 2019a) using the MSFragger-based FragPipe computational platform (Kong et al. 2017; Yu et al. 2020). Our reference proteome database contains highly specific protein sequences that increase the likelihood of unique peptide mapping, in contrast to some publicly available databases where the high degree of homology between proteins in the database may cause multiple mapping of peptides to proteins. Contaminants and reverse decoys were added to the database automatically. Carbamidomethylation of cysteine was selected as a fixed modification and oxidation of methionine was selected as a variable modification. The precursor-ion mass tolerance and fragment-ion mass tolerance were set at 10 and 12 ppm, respectively. Up to 2 missed tryptic cleavages were allowed and the protein-level false discovery rate (FDR) was set to <1%.

### ATAC-seq Data Generation, Processing, and Analysis

ATAC-seq data were generated for right venom gland tissue samples by Active Motif (Carlsbad, California), derived from snap-frozen glands of the same animals used for mRNA-seq. Raw ATAC-seq reads were mapped to the *C. viridis* reference genome using the “mem” algorithm from bwa v0.7.17 with default settings (Li 2013). Procedures for ATAC-seq data processing were largely based on an existing set of methods laid out in Perry et al. (2022). Briefly, PCR duplicates were removed using Picard Tools v2.22.6 (broadinstitute.github.io/picard), and samtools v1.9 (Li et al. 2009) was used to remove all nonunique alignments and improperly paired reads. The “randsample” command from MACS2 v2.2.7.1 (Zhang et al. 2008) was used to randomly down-sample reads to the number of tags present in the sample with the fewest tags. ATAC-seq peaks were called using MACS2 with a q-value cutoff of 0.001. To assess ATAC-seq data quality, we calculated the fraction of reads in peaks (FRiP) for each sample using featureCounts (Liao et al. 2014). Two ATAC-seq samples (a mid-latitude *C. viridis* (CV1081) and the *C. o. lutosus* (CV0987) individual) were excluded from subsequent ATAC-seq analyses due to low FRiP scores (supplementary table S1, Supplementary Material online). The “merge” command from bedtools v2.29.2 (Quinlan and Hall 2010) was used to merge partially overlapping peak regions between two or more samples. This set of merged peak regions was used for downstream analyses. Bigwig files of raw read coverage in each sample were generated using the “bamCoverage” command in deepTools v3.1.3 (Ramírez et al. 2016) with a bin size of 32 bp. The “multiBigwigSummary” command with options “–BED” and “–outRawCounts’ was then used to output a length-normalized average ATAC-seq signal matrix for the merged peak set. edgeR v3.32.1 in R was used to calculate TMM normalization factors for all samples, and these factors were then used to generate normalized bigwig files again using the “bamCoverage” command in deepTools. These processed, normalized bigWig files were used to produce ATAC-seq read depth tracks in R using the ggcoverage (Song and Wang 2023) package in R.

### Generation and Analysis of Genome resequencing Data

High coverage, re-sequenced genomes for the 12 samples (supplementary table S1, Supplementary Material online) used for prior analyses were also generated. DNA was extracted from snap-frozen blood using a phenol–chloroform–isoamyl (Invitrogen Life Technologies, No. 15593031) extraction protocol. Libraries were prepared from the DNA elution using Illumina Nextera Flex kits which were then sequenced on an Illumina NovaSeq 6000 using 150 bp paired-end reads, targeting an average coverage of 50X. Reads were filtered using Trimmomatic with the same settings specified above. These reads were then mapped to the reference genome using “bwa” at a mean unique read mapping rate of 97.93% and a mean coverage of 50.2X (supplementary table S1, Supplementary Material online).

Methods for variant calling and filtering were performed following methods previously described (Schield et al. 2022). Briefly, individual genomic variants were called using the “HaplotypeCaller” command from GATK v4.0.8.1 (McKenna et al. 2010) following best practices recommendations, and the resulting individual genomic variant call format (gVCF) files were then combined with the “CombineGVCF” command. The cohort gVCF was hard filtered based on GATK's parameter threshold recommendations with the “VariantFiltration” and “SelectVariants’ commands, which resulted in 17,051,557 variants.

Variants from the gVCF were projected onto the reference venom CRE sequences using the “consensus’ command from bcftools v1.16 (Danecek and McCarthy 2017) to produce individual variant sequences for each venom CRE. These variants were also checked for coverage depth, and base call error (supplementary table S3, Supplementary Material online). The reference CRE sequences used here were obtained from a prior study that investigated venom regulatory architecture in *C. viridis* by integrating multiple functional genomics approaches (including ChIP-seq, ATAC-seq, and chromatin contact data) to assign venom genes to genomic regions (Perry et al. 2022). Nucleotide diversity (π) from these sequence files was calculated using a custom R script, in which consensus sequences were first aligned using muscle (Edgar 2004). To identify variants that were found only in *C. viridis,* the gVCF was filtered using bcftools “filter” command to retain variants where all *C. viridis* samples contain the reference allele or the alternate allele, but non-*C. viridis* samples contain the opposite, respectively.

### TFBS Scanning and Footprinting Analyses

The JASPAR 2022 non-redundant vertebrate motif database (Castro-Mondragon et al. 2022) was subset to retain the 161 TFs of interest with respect to venom gene regulation from a prior study (Perry et al. 2022) as well as TFs differentially expressed between *C. viridis* and non-*C. viridis* from venom mRNA-seq data, described above. The individual variant CRE sequence files described above were concatenated and scanned for TFBSs with this custom JASPAR motif set with the “scan” option in Ciiider v0.9 (Gearing et al. 2019), using the default motif similarity threshold of 0.15. Differential binding was assessed using ATAC-seq footprinting analysis, which was performed using TOBIAS v.0.12.4 (Bentsen et al. 2020) following the methods described in a prior study (Perry et al. 2022). Briefly, insertion site bias was corrected using the “ATACorrect” command, footprint scores were calculated using “ScoreBigwig” and “BINDetect” was used to calculate sample-specific footprint score binding thresholds. The sample-wide set of scanned TFBS regions was used as input to deepTools “multiBigwigSummary”, using the same options described above, to produce a sequence length-normalized matrix of ATAC-seq scores at all TFBSs which were then binarized using the binding threshold to contrast bound and unbound TFBSs per individual. VCF variants were then intersected with the bound TFBSs with a custom R script to assess differentially bound TFBSs which contain variants at the motif.

### Exploring Evidence of Evolutionary Correlates With Venom Expression Variation

Input feature tables for linear modeling were constructed per venom gene by assembling datasets as follows. For each individual, tables contained DeSEQ2-normalized gene expression for the gene of interest, accessibility scores at peaks falling within promoters and/or enhancers for that gene, accessibility scores at peaks containing loci bound by CTCF (Perry et al. 2022) which fall within a window defined as a ± 1 kb extension around the furthest separated features of a venom gene array (i.e. known CREs or coding regions), accessibility at the top three non-CRE, non-CTCF associated peaks with the highest variation in ATAC-seq scores within the same venom array windows defined above, binarized footprints for venom-regulating TFs binding TFBSs in CREs for the gene (‘0' = TF is not bound at TFBS in that sample, “1' = TF is bound at TFBS in that sample), DeSEQ2-normalized expression for all venom-regulating TFs, and numerically recoded genotypes (‘0' = homozygous reference, “1' = heterozygous, “2' = homozygous alternate) for variants that occur within CREs of that venom gene.

A guide species tree for phylogenetic PCA was obtained from a prior study (Schield et al. 2019b), and middle, southern, and northern latitude *C. viridis* populations were collapsed. All original feature values were first transformed into phylogenetically independent contrasts using the “pic” function from the ape package (Paradis et al. 2004) to account for shared covariance among the species (Felsenstein 1985). We explored for evidence of evolutionary correlates with venom expression variation using principal component regression based on phylogenetic independent contrasts computed for each set of features. Specifically, we used this approach to evaluate whether variation across regulatory features (and classes of features) were correlated with venom expression according to the classes of input predictor features described above. Where the number of measured variables exceeded the number of samples, PCA of the phylogenetically corrected features was performed to obtain the first principal axis (PC1) for that feature class; these components were subsequently used as input predictor variables for multiple regression using the phylogenetic independent contrasts of normalized venom expression as the response variable. PCAs were conducted for the phylogenetic contrasts of each feature using the “prcomp” base function in R, and linear models were fit using the “lm” function.

## Supplementary Material

evae110_Supplementary_Data

## Data Availability

ATAC-seq, RNA-seq, and whole-genome resequencing data generated in this study has been submitted to the NCBI under BioProject accession PRJNA1061517. Processed ATAC-seq data have also been submitted to NCBI's Gene Expression Omnibus, under accession GSE254420. Code and additional data for reproducing main text figures and key analyses are available at github.com/SidG13/CrotalusVenomFxnGenomics. Any additional information required to reanalyze the data reported here is available upon request to the Author for Correspondence.
