# Supplementary material for: Diverse Gene Regulatory Mechanisms Alter Rattlesnake Venom Gene Expression at Fine Evolutionary Scales
Source: Genome Biol Evol. 2024 May 16;16(7):evae110. doi: 10.1093/gbe/evae110 (PMC11243404; doi:10.1093/gbe/evae110)
Supplement: evae110_Supplementary_Data [file evae110_supplementary_data.zip › _VenomPopFxn_Figures_trimmed_ONLY_SUPPLEMENT_2024.03.27.pdf]

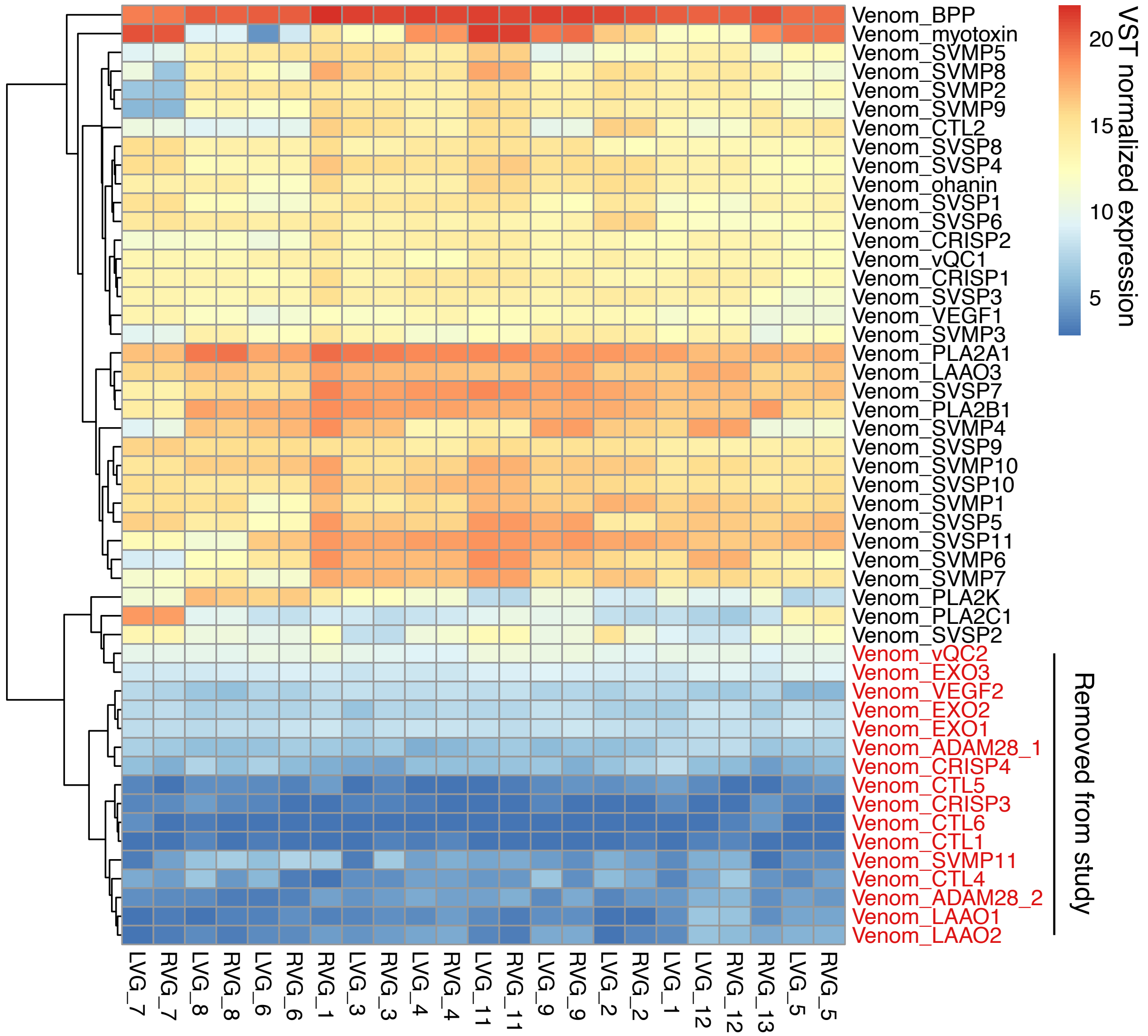

Supplementary Figure 1. Heatmap of venom gene (N=50) expression across venom gland samples. Venom genes with low expression in all venom gland samples (N = 16) were entirely removed from the study. The removed genes are indicated with a black bar and highlighted with red text.

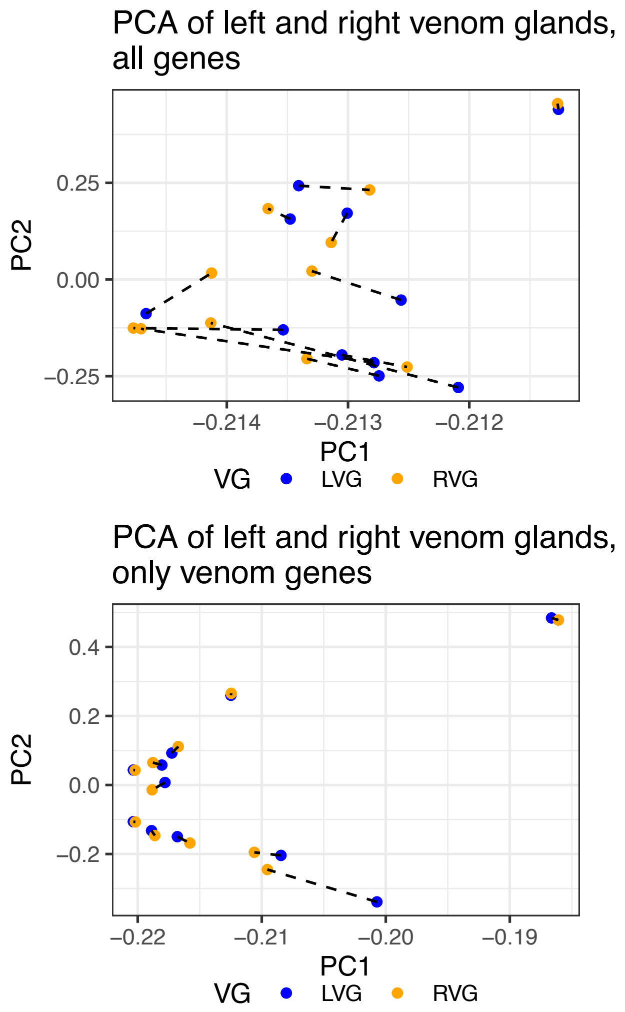

Supplementary Figure 2. PCA of mRNA expression between left and right venom glands (LVG and RVG) for all genes (top) and only venom genes (bottom). Expression variation is greatly minimized when considering the expression of only venom genes. The dashed line connects LVG and RVG from the same individual.

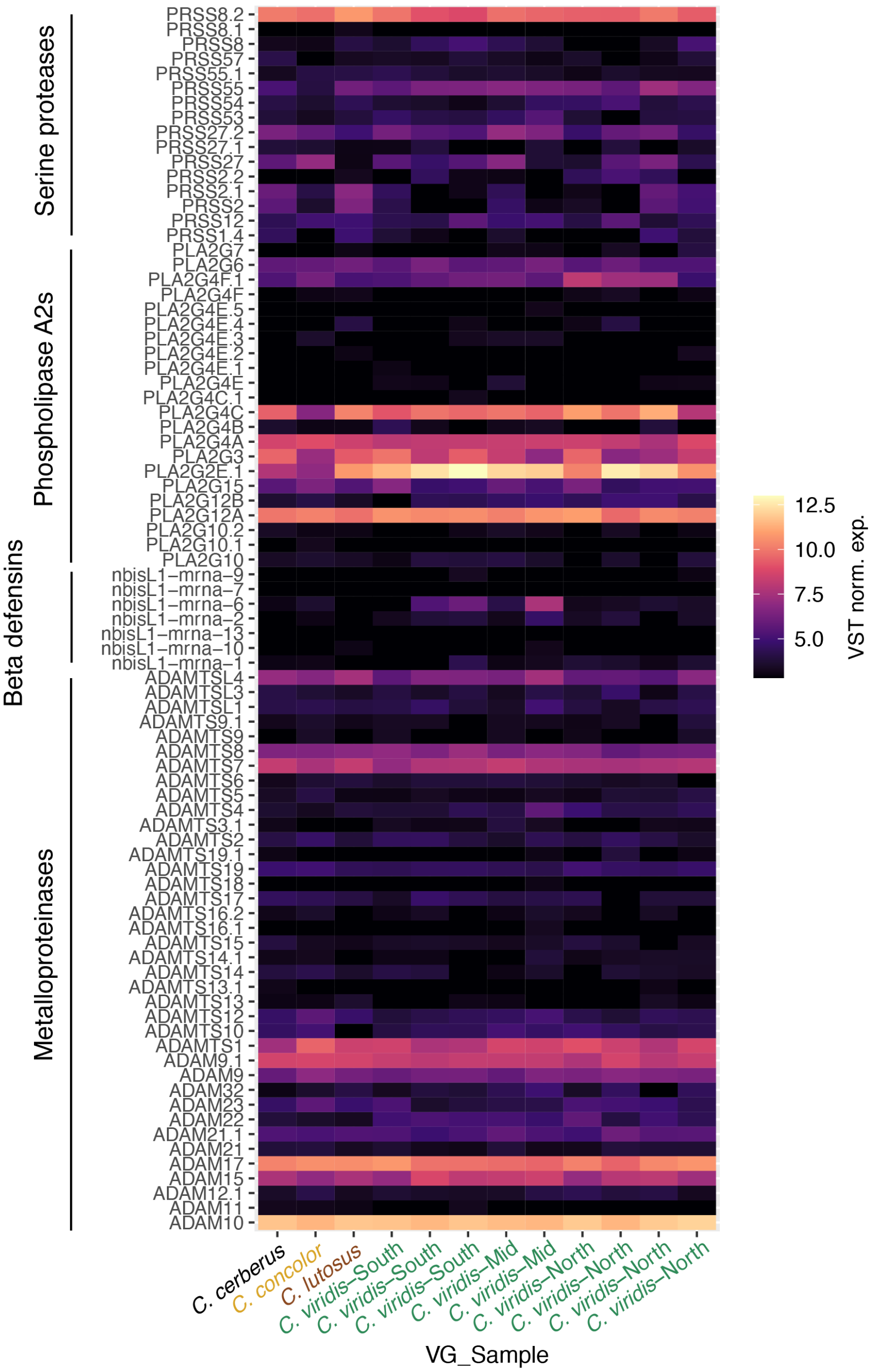

Supplementary Figure 3. Heatmap of non-venom paralog expression averaged across left and right venom glands per samples. Non-venom paralogs were selected based on membership to specific families, shown to the left.

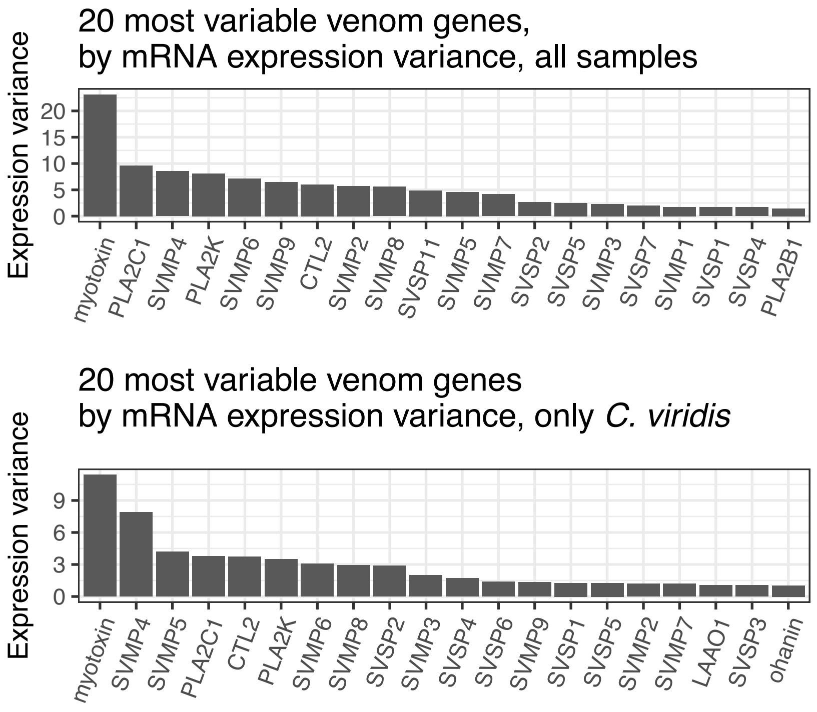

Supplementary Figure 4. Top 20 most variable venom genes considering all samples (including non *Crotalus viridis* lineages), and only *C. viridis* samples.

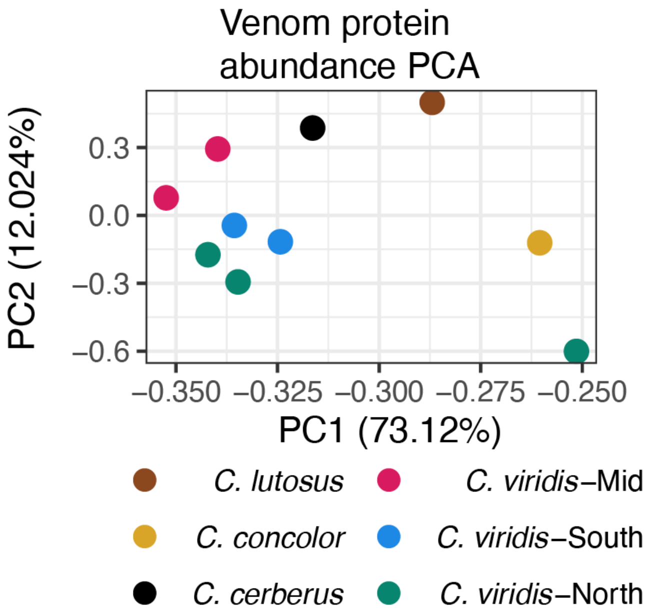

Supplementary Figure 5. PCA of venom protein expression across samples.

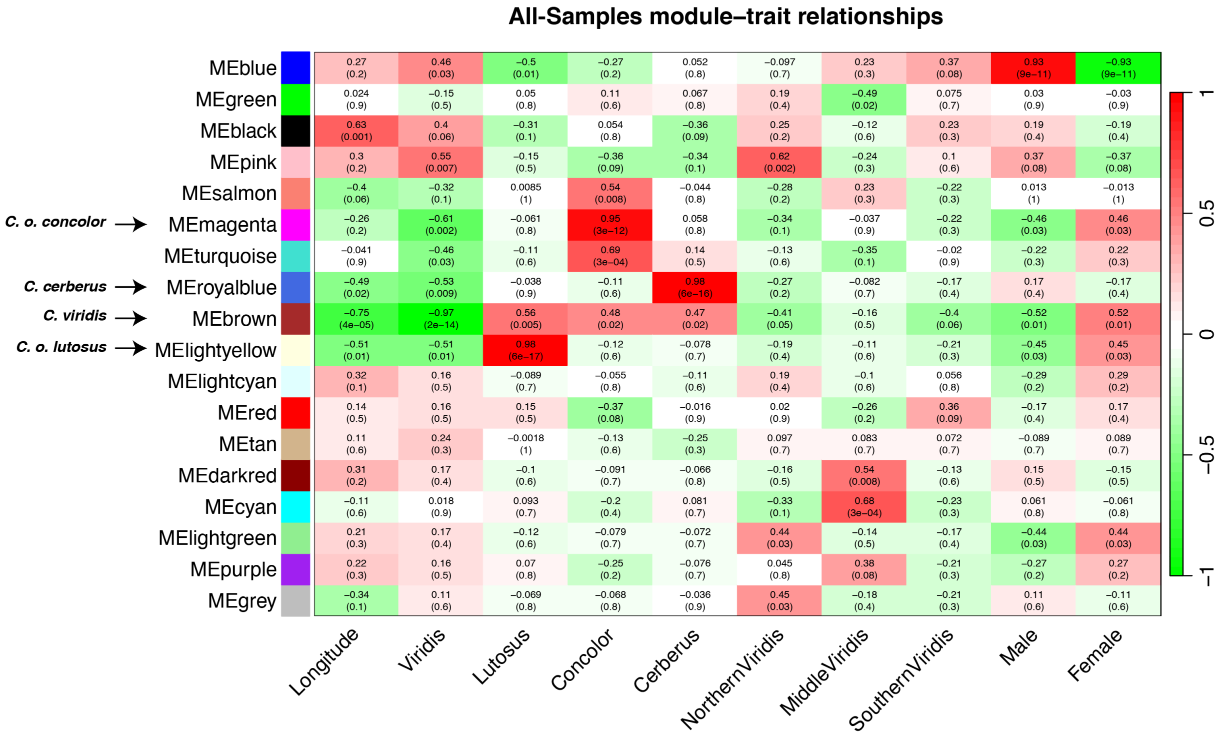

Supplementary Figure 6. Module-trait significance matrices from WGCNA when running with all samples, including non-*C. viridis* individuals (N=23). Sample traits are coded both as continuous variables (longitude), and categorical variables (species, *C. viridis* population, and sex). From the all-sample run, the four species identity traits were most significant for the following modules: *C. viridis* ("brown"), *C. o. lutosus* ("lightyellow"), *C. o. concolor* ("magenta") and *C. cerberus* ("royalblue").

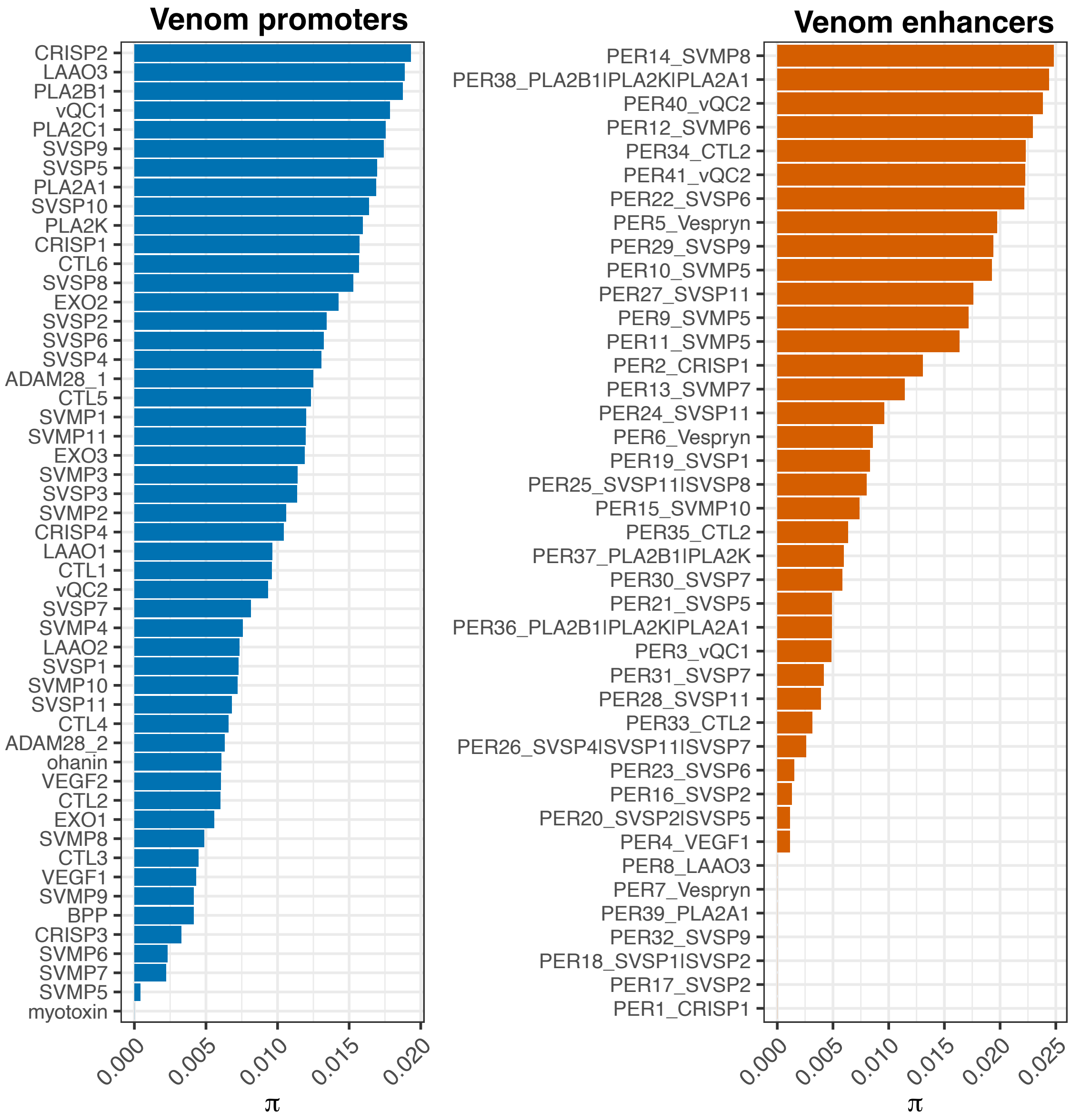

Supplementary Figure 7. Nucleotide diversity estimates for venom gene cis-regulatory elements (CREs). For enhancers, the venom gene is shown after the underscore. Pipes are used to separate genes influenced by multigene enhancers. Note several CREs have 0 variation, particularly some SVSP enhancers and the myotoxin promoter.

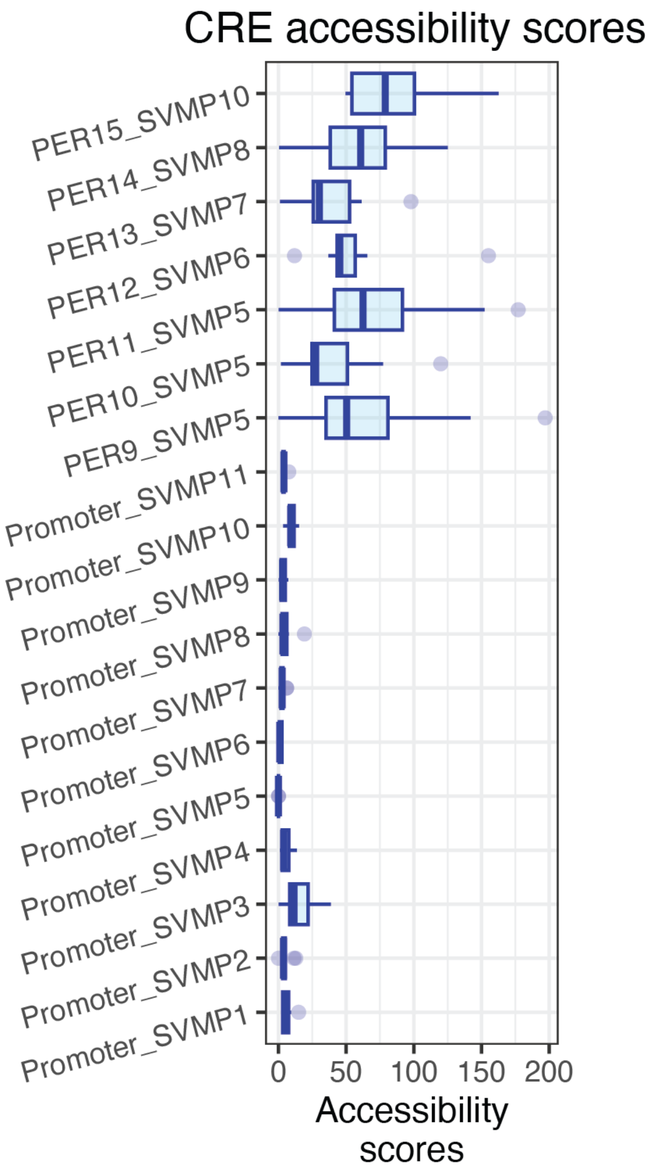

Supplementary Figure 8. Chromatin accessibility scores at cis-regulatory elements (promoters and enhancers) of SVMP paralogs.

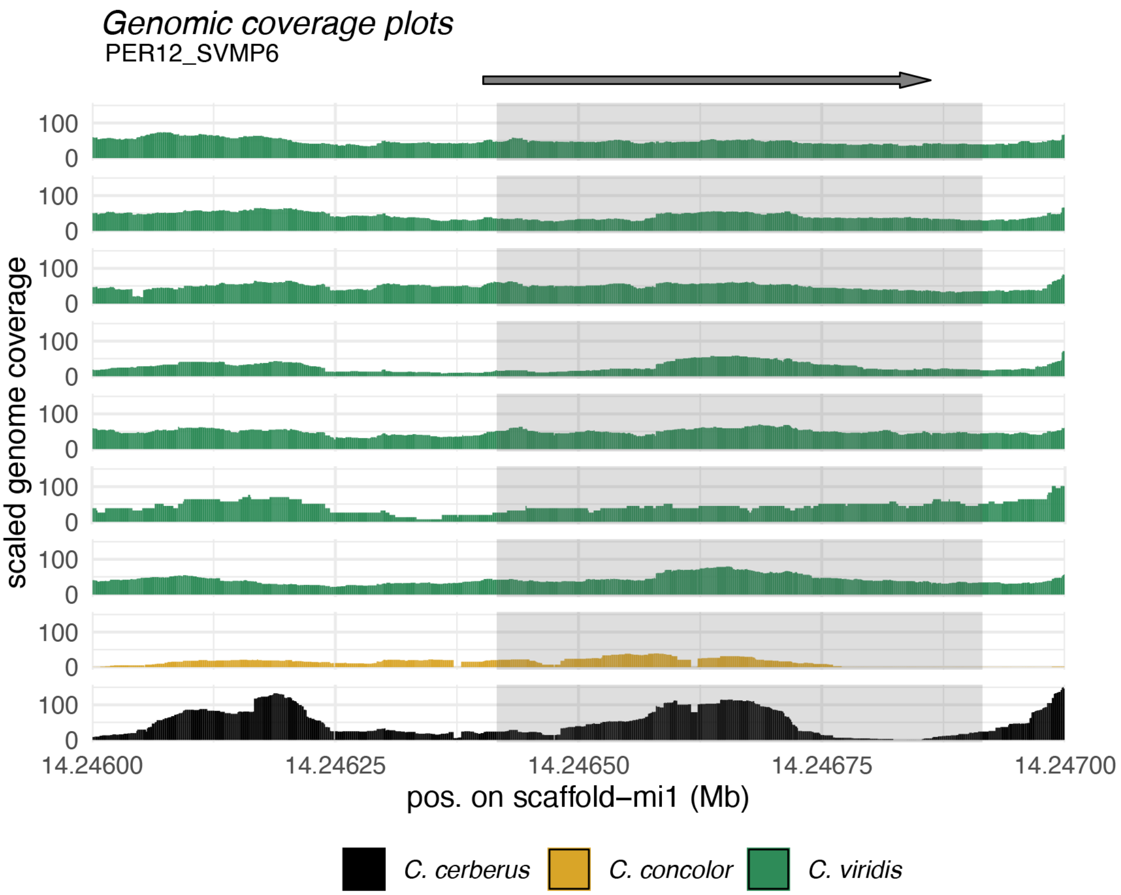

Supplementary Figure 9. Genomic read depth coverage of individuals at the SVMP6 enhancer showing no major differences in coverage at the enhancer. The grey arrow represents the location of the enhancer, and the grey rectangles represent consensus peaks called from ATAC-seq data data that overlap the enhancer. Individual coverage track scores have been scaled so each track has the same average depth.

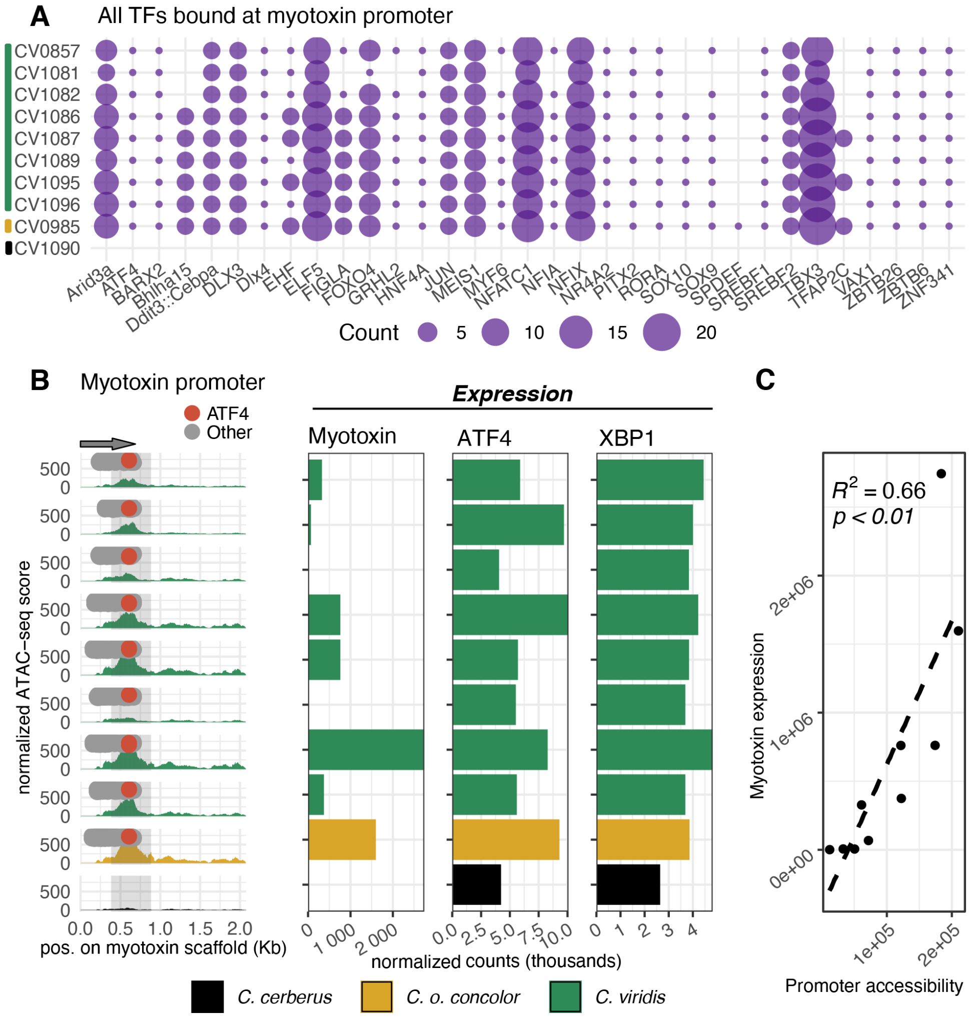

Supplementary Figure 10. Regulatory landscape at the myotoxin promoter. (A) There is predictably low overall TF occupancy variation across samples, given the lack of nucleotide variation at the myotoxin promoter. (B) There is evidence of the UPR-associated TF ATF4 binding to the promoter of myotoxin, which was suggested through transcriptomic-based correlations. Though evidence of XBP1 binding was not detected. (C) Accessibility at the promoter correlates strongly with myotoxin expression.

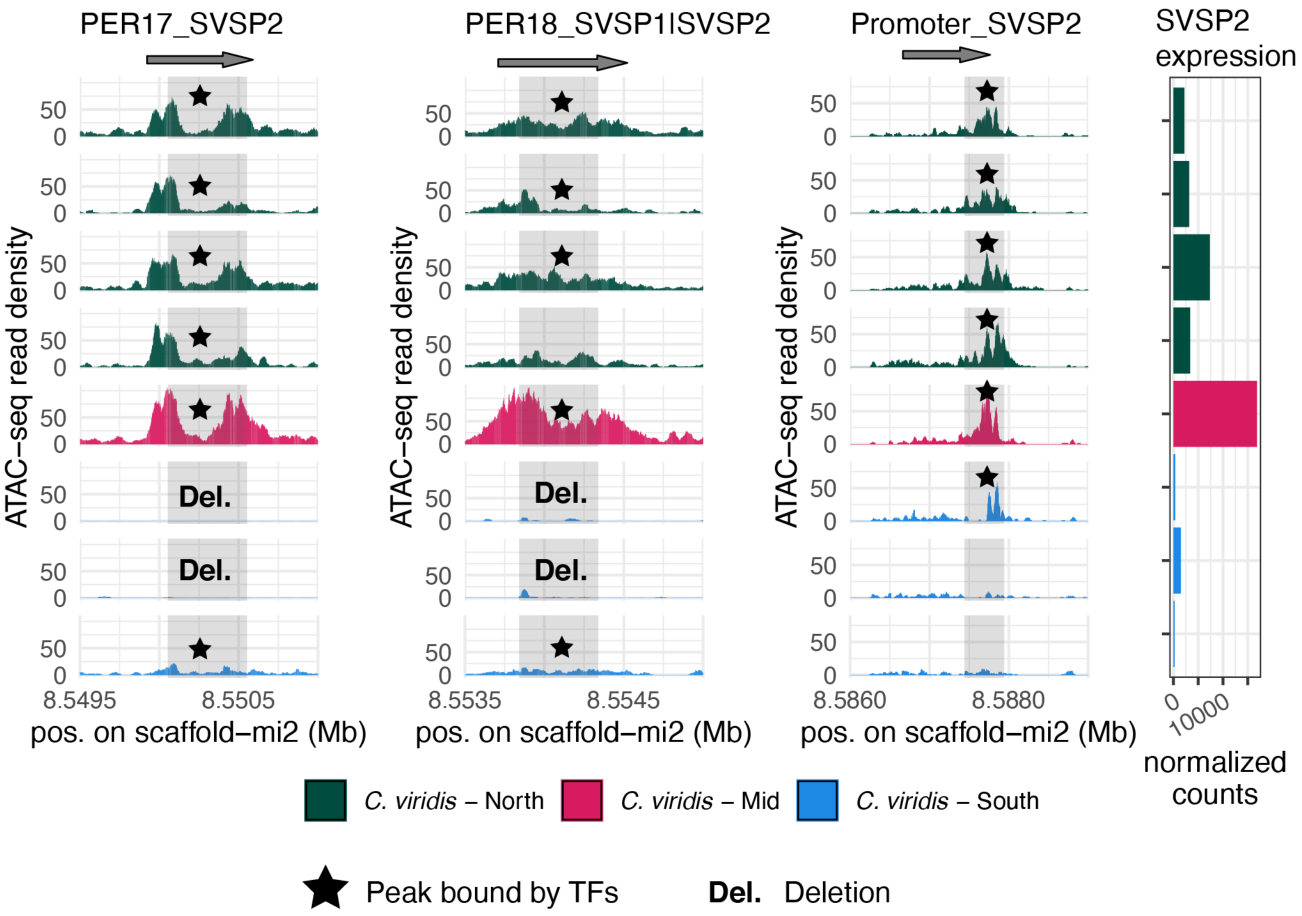

Supplementary Figure 11. ATAC-seq coverage of *C. viridis* individuals at SVSP2 CRE regions (PER17, PER18 and SVSP2 promoter). SVSP2 expression in DESeq2 normalized counts are shown in the right-most plot. The loci denoted with “Del.” indicate evidence of a large deletion affecting the locus. The grey arrows represents the location of the CREs, and the grey rectangles represent consensus peaks called from ATAC-seq data that overlap the CREs. Black stars represents the CRE is predicted to be bound by TFs.

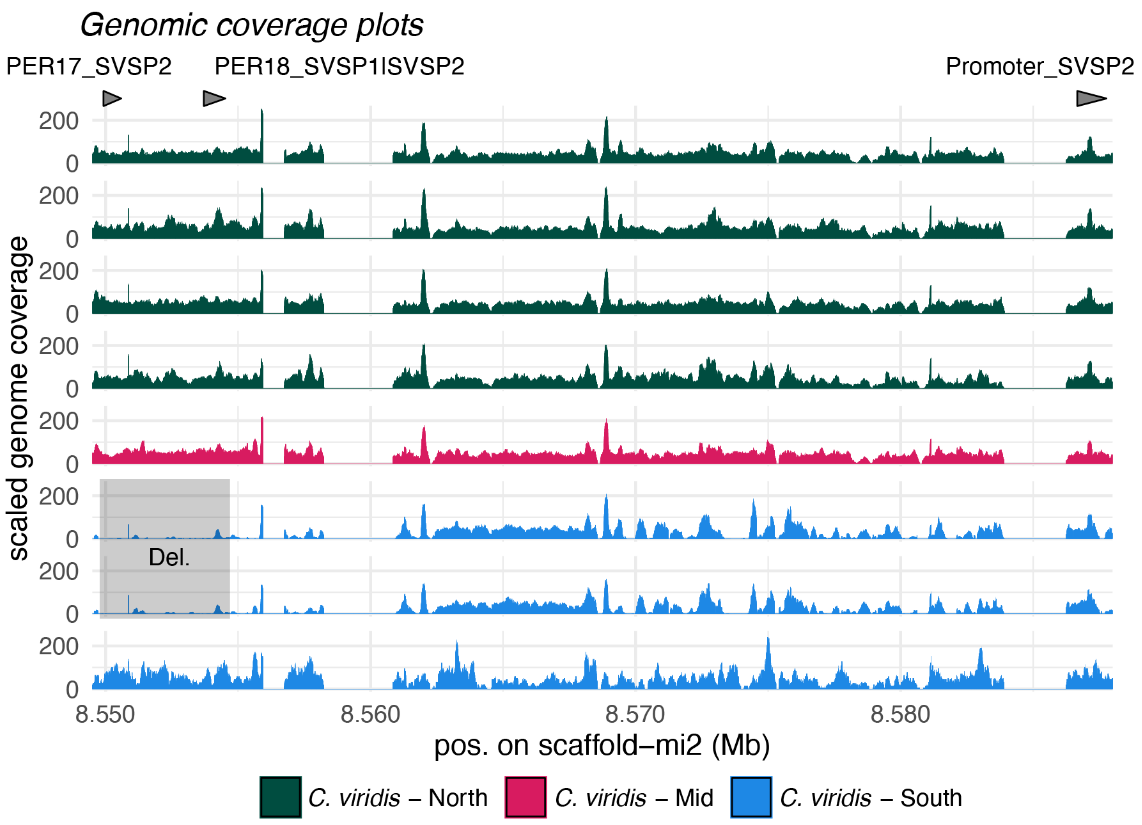

Supplementary Figure 12. Genomic read coverage of *C. viridis* individuals at SVSP2 CRE regions (PER17, PER18 and SVSP2 promoter). The order of individuals displayed matches that of Supplementary Figure 11. The locus denoted with "Del." indicate evidence of a large deletion affecting the locus, relative to other individuals. The grey arrows represents the location of the CREs. Individual coverage track scores have been scaled by library size.
